# Defect‐Induced Dense Amorphous/Crystalline Heterophase Enables High‐Rate and Ultrastable Sodium Storage

**DOI:** 10.1002/advs.202205575

**Published:** 2022-10-30

**Authors:** Sahar Osman, Chao Peng, Fangkun Li, Haoliang Chen, Jiadong Shen, Zeming Zhong, Wenjie Huang, Dongfeng Xue, Jun Liu

**Affiliations:** ^1^ School of Materials Science and Engineering and Guangdong Provincial Key Laboratory of Advanced Energy Storage Materials South China University of Technology Guangzhou Guangdong 510641 China; ^2^ Multiscale Crystal Materials Research Center Shenzhen Institute of Advanced Technology Chinese Academy of Science Shenzhen 518055 China

**Keywords:** amorphous/crystalline heterophase, defect‐rich, lattice distortion, oxygen vacancy, sodium‐ion batteries, vanadium trioxide

## Abstract

Currently, the construction of amorphous/crystalline (A/C) heterophase has become an advanced strategy to modulate electronic and/or ionic behaviors and promote structural stability due to their concerted advantages. However, their different kinetics limit the synergistic effect. Further, their interaction functions and underlying mechanisms remain unclear. Here, a unique engineered defect‐rich V_2_O_3_ heterophase structure (donated as A/C‐V_2_O_3−_ *_x_* @C‐HMCS) composed of mesoporous oxygen‐deficient amorphous _−_ hollow core (A‐V_2_O_3−_ *_x_*/HMC) and lattice‐distorted crystalline shell (C‐V_2_O_3_/S) encapsulated by carbon is rationally designed via a facile approach. Comprehensive density functional theory (DFT) calculations disclose that the lattice distortion enlarges the porous channels for Na^+^ diffusion in the crystalline phase, thereby optimizing its kinetics to be compatible with the oxygen‐vacancy‐rich amorphous phase. This significantly reduces the high contrast of the kinetic properties between the crystalline and amorphous phases in A/C‐V_2_O_3−_ *_x_* @C‐HMCS and induces the formation of highly dense A/C interfaces with a strong synergistic effect. As a result, the dense heterointerface effectively optimizes the Na^+^ adsorption energy and lowers the diffusion barrier, thus accelerating the overall kinetics of A/C‐V_2_O_3−_ *_x_* @C‐HMCS. In contrast, the perfect heterophase (defects‐free) A/C‐V_2_O_3_@C‐HCS demonstrates sparse A/C interfacial sites with limited synergistic effect and sluggish kinetics. As expected, the A/C‐V_2_O_3−_ *_x_* @C‐HMCS achieves a high rate and ultrastable performance (192 mAh g^−1^ over 6000 cycles at 10 A g^−1^) when employed for the first time as a cathode for sodium‐ion batteries (SIBs). This work provides general guidance for realizing dense heterophase cathode design for high‐performance SIBs and beyond.

## Graphical abstract

A defect‐rich heterophase structure A/C‐V_2_O_3−_ *_x_* @C‐HMCS composed of oxygen‐deficient amorphous hollow core and lattice‐distorted crystalline shell encapsulated by carbon is synthesized via a novel strategy. The defects significantly reduce the high contrast of the kinetic properties between the crystalline and amorphous phases and induce the formation of dense A/C interfaces, lowering the energy barriers, and enabling fast and stable SIBs performance.

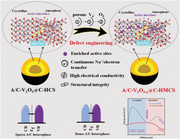

## 1 Introduction

In recent decades, rechargeable lithium‐ion batteries (LIBs) have witnessed great success in electric vehicles and portable electronic due to their high energy density and long cycle life.^[^ ^1^, ^2^ ^]^ However, the uneven global distribution of lithium resources and ever‐increasing price, hinder their large‐scale applications.^[^ ^3^, ^4^ ^]^ Sodium‐ion batteries (SIBs) have emerged as a prominent alternative due to the abundance and low cost of sodium resources and their similar working principles to lithium‐ion batteries.^[^ ^5^, ^6^ ^]^ Nevertheless, the drastic volume expansion and sluggish reaction kinetics caused by the large Na^+^ radius (1.02 Å) inhibit its development.^[^ ^7^, ^8^ ^]^ Therefore, it is critical to customize cathode materials for SIBs to better accommodate the bulkier Na‐ions. Vanadium oxides have been intensively explored as one of the most promising cathodes due to their multiple valence states (V^2+^‐V^+5^), appropriate operating voltages, high theoretical capacities, and facile synthetic chemistry.^[^ ^9^, ^10^, ^11^ ^]^ However, their oxidation state plays a significant role in their electrochemical properties. For example, vanadium pentoxide with a higher valence (V^+5^) shows a high theoretical capacity of 442 mAh g^−1^, which is much larger than currently used commercial cathodes.^[^ ^12^ ^]^ Further, various V_2_O_5_ nanostructures have been widely reported to improve their electrochemical performance.^[^ ^11^, ^13^ ^]^ Unlike other vanadium oxides, vanadium trioxide V_2_O_3_ with a low valence state (V^3+^) has been considered unusable as a cathode for lithium/sodium‐ion batteries by Tranchant et al. in 1980.^[^ ^14^ ^]^ Nevertheless, it demonstrates great potential as an anode for Li/Na storage.^[^ ^15^, ^16^, ^17^ ^]^

Notably, most of the reported cathodes are crystalline frameworks in which Na^+^ intercalates into certain active sites and diffuses along specific paths, thus limiting the specific capacity and overall reaction kinetics. Worse still, the bulk framework will be pulverized during the repeated Na^+^ intercalation/deintercalation process due to variation of stresses, leading to poor cycling stability.

To solve the above issues, much attention has been devoted to the amorphization strategy.^[^ ^18^, ^19^ ^]^ In comparison, isotropic amorphous materials with abundant porous channels and unsaturated coordination sites provide extra Na^+^ storage sites and improve Na^+^ binding ability, ensuring rapid intercalation kinetics compared to their crystalline counterpart.^[^ ^18^, ^19^, ^20^ ^]^ Besides, amorphous materials with characteristic open frameworks allow only small volume expansion during sodiation/desodiation process, which is beneficial for long‐term cyclability. However, their inherently low electrical conductivity reduces the electrochemical activity.^[^ ^21^, ^22^ ^]^ Recently, many researchers have reported that the construction of defects in amorphous metal oxides such as vacancies can boost intrinsic electronic conductivity and facilitate charge transport.^[^ ^19^, ^23^, ^24^ ^]^ Nevertheless, the complex and unscalable synthesis limits their applications.^[^ ^25^ ^]^

Hence, a promising cathode design for sodium storage should balance high stability and fast kinetics by harmonizing the individual advantages of crystalline and amorphous phases. To achieve this, the construction of the amorphous/crystalline A/C heterostructure is an ideal strategy to improve the electrochemical performance of SIBs, as they tend to have better physicochemical properties than their single counterparts.^[^ ^26^, ^27^ ^]^ The abundant unsaturated coordination sites of the amorphous phase offer plentiful active sites while the crystalline phase boosts electron transfer. In addition, the heterointerfaces A/C can significantly optimize the adsorption/desorption of ions, as well as, electron densities of the interfacial sites, thereby facilitating the charge transfer during repeated cycles.^[^ ^28^ ^]^ Further, the structural stability can be enhanced due to the synergistic effect between different phases.^[^ ^29^ ^]^ Despite these promising prospects, the fabrication of such amorphous/crystalline heterostructure electrodes remains to be a great challenge. Furthermore, most reported heterophase materials are either heterogeneous, in which the crystalline and amorphous interfaces consist of different chemical compositions, such as Co_3_O_4_ (crystalline)/TiO_2_ (amorphous),^[^ ^30^ ^]^ or homogeneous materials with the sparse crystalline and amorphous interfaces, such as Nb_2_O_5_, Si, and SnO_2_.^[^ ^26^, ^31^, ^32^ ^]^ Unfortunately, their cycle life has been so far limited to less than 1000 cycles. Further, how to rationally design and controllably synthesize well‐defined crystalline/amorphous heterophase with similar chemical components remains a major challenge. Further, there is still a lack of powerful theoretical guidance for the design of high‐performance heterophase electrodes, and its interaction functions and underlying mechanisms still seem blind.

**Figure 1:**
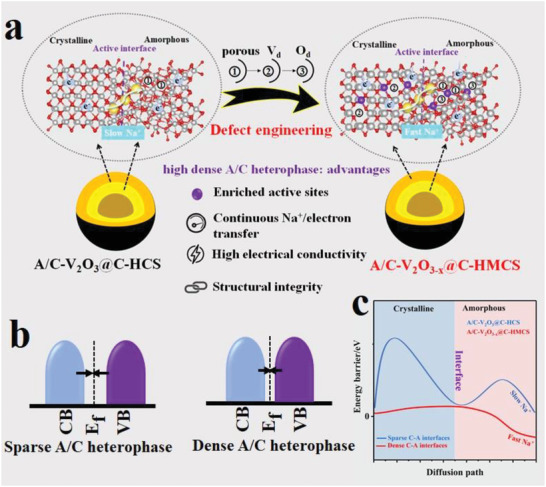
Schematic illustration of interfacial defect engineering and the electrochemical advantages of high‐density heterointerfaces. a) Model of pure A/C‐V_2_O_3_@C‐HCS and defect‐rich porous structure A/C‐V_2_O_3−_ *_x_* @C‐HMCS, b) bandgap, and c) diffusion path.

Herein, we report the design of a unique engineered defect‐rich V_2_O_3_ amorphous/crystalline heterophase structure (donated as A/C‐V_2_O_3−_ *_x_* @C‐HMCS) composed of oxygen‐deficient amorphous V_2_O_3−_ *_x_* hollow mesoporous core (A‐V_2_O_3−_ *_x_*/HMC) and lattice‐distorted crystalline V_2_O_3_ shell (C‐V_2_O_3_/S) encapsulated by carbon, which is successfully prepared via a one‐pot hydrothermal method followed by a reduction reaction and activation process (**Figure** **1**a). Based on DFT calculations, in pristine heterophase (defects‐free) A/C‐V_2_O_3_@C‐HCS, the adsorption energy is significantly reduced at the heterointerface sites due to the large difference in kinetics properties between the crystalline and amorphous phases, indicating that the sparse interfacial sites limit the optimization of adsorption energy, thus sluggish Na^+^ kinetics. In contrast, the defective heterophase A/C‐V_2_O_3−_ *_x_* @C‐HMCS demonstrates the strongest Na^+^ adsorption energy in either the crystalline phase, amorphous phase, or even at heterointerfaces sites, indicating that the defects induce the formation of highly dense A/C interfacial sites, which can not only modulate the electronic structure and enrich active sites, but also lowers the energy barriers, and promote adsorption energies, thus rapid Na^+^ kinetics (Figure 1b,c). In particular, tailoring the unsaturated active site is an effective strategy to promote charge transfer. In addition, the ex‐HRTEM and ex‐XRD results demonstrate that the dense A/C interfacial sites can be well maintained even after long‐term cyclability, further confirming the robust stability of A/C‐V_2_O_3−_ *_x_* @C‐HMCS. Benefiting from these merits, the A/C‐V_2_O_3−_ *_x_* @C‐HMCS exhibits superior electrochemical properties when employed for the first time as a cathode for sodium storage in the range of 1.5–4.0 V: a high capacity of 261 mAh g^−1^ is achieved at a low rate of 0.1 A g^−1^. Even at a high rate of 10 A g^−1^, the A/C‐V_2_O_3−_ *_x_* @C‐HMCS cathode still retains a high capacity of 192 mAh g^−1^ over 6000 cycles, outperforming most of the reported vanadium‐based cathodes for SIBs. These results evidenced that the electrochemical properties of electrode materials depend not only on the oxidation state but also largely on their crystal structure and morphology. Further, this work sheds light on promising dense heterophase material design for next‐generation SIBs from both an experimental and theoretical perspective.

## 2 Results and Discussion

### 2.1 Synthesis and Characterizations

**Figure 2:**
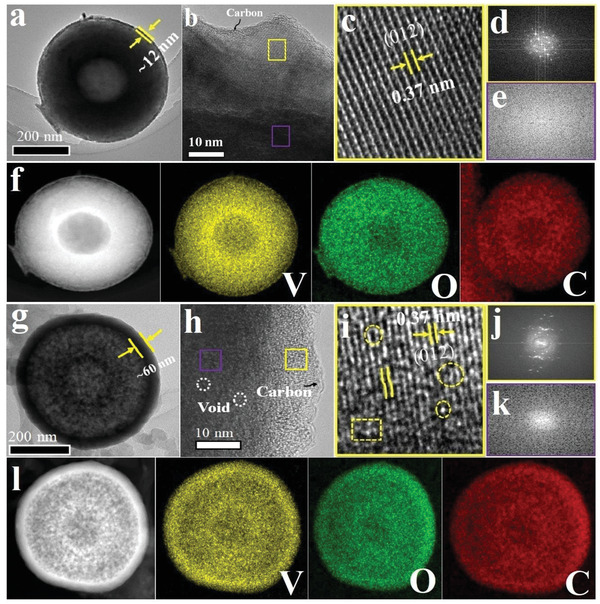
Morphology and characterizations of A/C‐V_2_O_3_@C‐HCS and A/C‐V_2_O_3−_ *_x_* @C‐HMCS. a,g) TEM; b,h) HRTEM images; c,i) inverse of fast Fourier transform (IFFT) patterns, the circles/squares indicate the defects and lattice deformation; FFT patterns are taken d,j) from the corresponding yellow squares and e,k) from violet squares areas in (b) and (h); and f, l) HAADF‐STEM and mapping images of A/C‐V_2_O_3_@C‐HCS and A/C‐V_2_O_3−_ *_x_* @C‐HMCS, respectively.

The synthesis process of the amorphous/crystalline V_2_O_3_‐based compounds core–shells encapsulated by carbon (denoted as A/C‐V_2_O_3_@C‐M) is schematically illustrated in Figure S1, Supporting Information. Typically, the V_2_O_5_ spheres precursor was prepared via a one‐pot solvothermal method as described in our previous report (Figure S2, Supporting Information).^[^ ^33^ ^]^ Its XRD profile shows only one diffraction pattern at 21° corresponding to the (001) plane of V_2_O_5_ (Figure S3, Supporting Information). First, the precursor was reduced at 500 °C under Ar/H_2_ for 2 h to obtain A/C‐V_2_O_3_@C‐CS (synthesis route in Figure S1, Supporting Information). The scanning electron microscopy (SEM) images show spheres with a diameter of ≈500 nm (Figure S4a,b, Supporting Information). A transmission electron microscopy (TEM) image demonstrates a like core–shell morphology with an average shell thickness of ≈80 nm (Figure S5a, Supporting Information). The high‐resolution TEM (HRTEM) image confirms its core–shell structure (Figure S5b, Supporting Information). The HRTEM of the core region shows no obvious lattice fringes, and its corresponding fast Fourier transform (FFT) pattern exhibits diffuse rings. In contrast, the HRTEM of the shell region demonstrates manifest interplanar spacing of 0.37 nm, which corresponds to the (012) plane of the rhombohedral V_2_O_3_. Further, its FFT patterns reveal bright spots, indicating that a typical core–shell is composed of an amorphous core and crystalline shell (Figure S5b1,b2, Supporting Information). In addition, the HRTEM also displays a thin layer of carbon. HADDF and its corresponding EDX mapping images verify that V, O, and C elements are evenly distributed on the microsphere's surface (Figure S5c, Supporting Information). Second, the A/C‐V_2_O_3_@C‐WCS sample was achieved by annealing of A/C‐V_2_O_3_@C‐CS for 2 h under N_2_ (see the synthesis route in Figure S1, Supporting Information). As shown in Figure S4c,d (Supporting information), the SEM images still reveal a spherical morphology with a smooth surface. The TEM image demonstrates a well‐defined core–shell structure, and the thickness of the crystalline V_2_O_3_ shell decreases from 80 to 60 nm (Figure S5d, Supporting Information). A clear interface has proved to be beneficial for long‐term cyclability.^[^ ^26^, ^33^, ^34^ ^]^ The HRTEM image (Figure S5e, Supporting Information) and its corresponding FFT patterns (Figure S5e1,2, Supporting Information) demonstrate the amorphous nature of the V_2_O_3_ core and the single crystallinity of the V_2_O_3_ shell, which are well consistent with the A/C‐V_2_O_3_@C‐CS. The corresponding EDX mappings images reveal that the elements of V, O, and C are distributed uniformly in the as‐prepared composite, verifying that the V_2_O_3_ core shell is coated by carbon (Figure S5f, Supporting Information). The hollow core–shell (HCS) has considerable merits, such as high specific surface area and mechanical robustness, favorable for rapid kinetics and structural stability.^[^ ^35^, ^36^ ^]^ To synthesize the heterophase V_2_O_3_ hollow core–shell (A/C‐V_2_O_3_@C‐HCS), 1.0 mL of distilled water was added dropwise to a pot of the V‐glycerate precursor (see the synthesis route in Figure S6, Supporting Information). The SEM images of the vanadium hydrate‐glycerate precursor demonstrate hollow sphere features (Figure S7a,b, Supporting Information). As shown in Figure S7c, only one diffraction pattern at 21° corresponds to the (001) plane of V_2_O_5_ is detected, consistent with Figure S3, indicating its low crystallinity.^[^ ^33^ ^]^ By direct calcination of this precursor at a higher temperature of 600 °C under Ar/H_2_ for 2 h, the A/C‐V_2_O_3_@C‐HCS is obtained. The SEM image shows hollow V_2_O_3_ spheres (Figure S8a,b, Supporting Information). Where the TEM image displays a hollow core–shell structure (**Figure** **2**a and Figure S9, Supporting Information). Similar to the A/C‐V_2_O_3_@C‐WCS, both the crystalline shell (≈12 nm) and amorphous core can be observed in the A/C‐V_2_O_3_@C‐HCS (Figure 2b,c and Figure S10, Supporting Information). Which were further confirmed by the bright spots (Figure 2d) and diffuse ring (Figure 2e) in the corresponding FFT patterns, respectively. Furthermore, the EDX mappings images exhibit the homogeneous distribution of carbon all over the V_2_O_3_ hollow core–shell (Figure 2f). Next, the defect‐rich heterophase V_2_O_3_ sample was obtained (A/C‐V_2_O_3−_ *_x_* @C‐HMCS) by annealing of A/C‐V_2_O_3_@C‐HCS at 600 °C under pure N_2_ for 2 h (see the synthesis route in Figure S6, Supporting Information). The SEM image shows a porous sphere (Figure S11a,b Supporting Information). The TEM image confirms it's a hollow mesoporous core–shell structure (Figure 2g and Figure S12, Supporting Information). The hollow porous structure can offer abundant active sites and shorten ion diffusion paths.^[^ ^37^ ^]^ Besides, the internal void space can synergistically accommodate the volume expansion of V_2_O_3_ during sodiation, which is beneficial to maintain a stable nanostructure.^[^ ^38^ ^]^ The thickness of the outer shell was measured to be 60 nm. The HRTEM image demonstrates amorphous mesoporous defect‐rich core A‐V_2_O_3−_ *_x_* (Figure 2h,k) and lattice‐distorted crystalline shell C‐V_2−_ *_x_* O_3_ as verified by the I/FFT patterns (Figure 2i,j, Figure S13, Supporting Information). The lattice distortion can enrich various pores and electron channels that facilitate electron conduction and ions diffusion.^[^ ^39^, ^40^ ^]^ The elemental mappings show homogeneous distribution of V, O, and C in the A/C‐V2O3_−_ *_x_* @C‐HMCS (Figure 2l).

**Figure 3:**
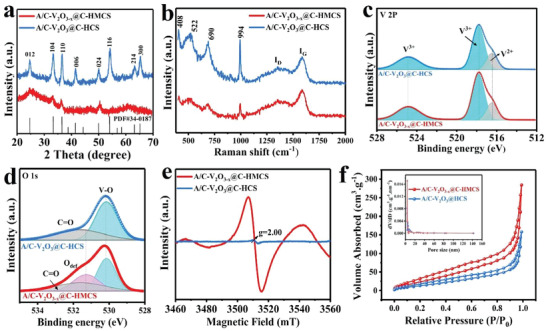
Phase, composition, and pore structure characterizations of A/C‐V_2_O_3_@C‐HCS, and A/C‐V_2_O_3−_ *_x_* @C‐HMCS. a) XRD pattern, b) Raman spectra, c) high‐resolution XPS spectra of V 2p, and d) O 1s. e) EPR spectra. f) N_2_ adsorption/desorption isotherms; inset, the corresponding pore size distribution.

To get further insight into the phase, composition, and pore structure of the as‐prepared A/C‐V_2_O_3_@C‐M core–shell nanostructures, various characterizations including X‐ray diffraction patterns (XRD), Raman spectrum, X‐ray Photoelectron Spectroscopy (XPS), and specific surface measurements were performed. All of these heterophase samples (A/C‐V_2_O_3_@C‐CS, A/C‐V_2_O_3_@C‐WCS, A/C‐V_2_O_3_@C‐HCS, and A/C‐V_2_O_3−_ *_x_* @C‐HMCS) are indexed to the rhombohedral V_2_O_3_ phase (JCPDS card no. 034–0187) (**Figure** **3**a, Figure S14a, Supporting Information).^[^ ^16^ ^]^ However, the A/C‐V_2_O_3−_ *_x_* @C‐HMCS displays only four broad diffraction peaks at 24.5°, 33.1°, 36.2°, and 50.1° assigned to (012), (104), (110), and (024) planes of V_2_O_3_, confirming the lattice distortion of the V_2_O_3_ crystalline shell, which is consistent with the HRTEM image (Figure 2i). To further investigate the characteristics of the samples, the results of the Raman spectra are depicted in Figure 3b and Figure S14b (Supporting Information). The Raman spectrum exhibits four peaks located at 408, 522, 690, and 994 cm^−1^, which corresponds to the V—O vibrations.^[^ ^15^ ^]^ Besides, it exhibits two broad peaks located at 1350.3 and 1586.5 cm^−1^, which are attributed to the disordered (D) and the graphene (G) bands of amorphous carbon, respectively, verifying that these samples were successfully encapsulated by carbon. The chemical and electronic surface states of the samples were examined by X‐ray photoelectron spectroscopy (XPS). For V 2p spectra in Figure 3c and Figure S14c (Supporting Information), the peaks are centered at 524.8 (V 2p_1/2_)/ 517.7 eV (V 2p_3/2_) and 516.4 (V 2p_3/2_) indicating the presence of V^3+^ and V^2+,^ respectively.^[^ ^41^ ^]^ The O 1s XPS spectra exhibit two peaks located at 530.6 and 532.0 eV, assigned to the V—O and C=O bonds, respectively (Figure 3d and Figure S14d, Supporting Information).^[^ ^41^ ^]^ An additional peak of the A/C‐V_2_O_3−_ *_x_* @C‐HMCS can be observed at 531.5 eV, indicating the formation of oxygen vacancy (O_V_).^[^ ^42^, ^43^ ^]^ C 1s spectra can be deconvolved into three peaks at 284.8, 285.7, and 288.8 eV and are assigned to the amorphous carbon C—C, C—O, and C=O bonds, respectively (Figures S14e and S15, Supporting Information).^[^ ^44^ ^]^ Electron paramagnetic resonance (EPR) was performed to identify oxygen vacancies in A/C‐V_2_O_3_@C‐HCS and A/C‐V_2_O_3−_ *_x_* @C‐HMCS (Figure 3e). The A/C‐V_2_O_3−_ *_x_* @C‐HMCS exhibits a distinct signal at *g* = 2.00, providing compelling evidence of oxygen vacancy formation and being well consistent with the XPS results.^[^ ^45^ ^]^ In addition according to the thermogravimetric analyses (TGA) (Figure S16, Supporting Information), the carbon contents of A/C‐V_2_O_3_@C‐CS, A/C‐V_2_O_3_@C‐WCS, A/C‐V_2_O_3_@C‐HCS, and A/C‐V_2_O_3−_ *_x_* @C‐HMCS were determined to be 15.50, 14.72, 14.03, and 16.75 wt%, respectively. Further, the specific surface areas and pore size distribution of the heterophase core–shell structures were measured by nitrogen adsorption‐desorption analysis (Figure 3f and Figure S14f Supporting Information). The A/C‐V_2_O_3−_ *_x_* @C‐HMCS sample displays a larger IV isotherm hysteresis loop compared to other samples, indicating its Meso‐sized porous structure. Based on the Brunauer–Emmett–Teller (BET) results, the specific surface area of A/C‐V_2_O_3−_ *_x_* @C‐HMCS (208.5 m^2^ g^−1^) is much higher than those of A/C‐V_2_O_3_@C‐HCS (151.4 m^2^ g^−1^), A/C‐V_2_O_3_@C‐WCS (75.0 m^2^ g^−1^), and A/C‐V_2_O_3_@C‐CS (63.0 m^2^ g^−1^). These structural merits, such as defect‐rich heterophase, larger specific surface area, and strong chemical interaction of the A/C‐V_2_O_3−_ *_x_* @C‐HMCS are believed to offer abundant active sites and various ion‐diffusion channels that accelerate electron/ion transfer, boost the structural stability, and facilitate the infiltration of electrolytes.

### 2.2 The First‐Principles Investigations

**Figure 4:**
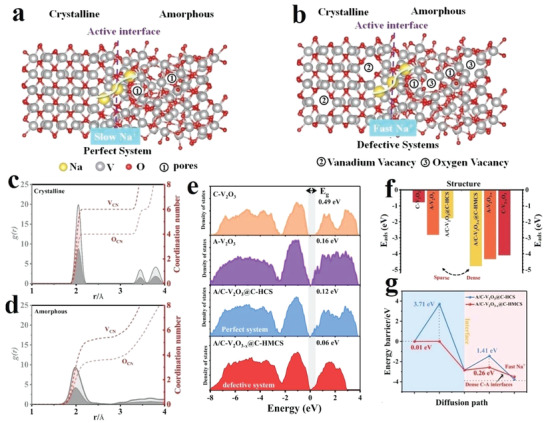
Structural illustration and DFT calculations: a) A/C‐V_2_O_3_@C‐HCS (perfect system) and b) A/C‐V_2_O_3−_ *_x_* @C‐HMCS (defective system). The coordination number of the c) crystalline V_2_O_3_ (C‐V_2_O_3_) and d) amorphous V_2_O_3_ (A‐V_2_O_3_). e) Comparison of the calculated electronic density of states (DOS) for C‐V_2_O_3_, A‐V_2_O_3_, A/C‐V_2_O_3_@C‐HCS, and A/C‐V_2_O_3−_ *_x_* @C‐HMCS. f) Adsorption energies and g) Na^+^ diffusion path and corresponding energy barrier profiles in A/C‐V_2_O_3_@C‐HCS and A/C‐V_2_O_3−_ *_x_* @C‐HMCS.

DFT calculations were conducted to investigate the Na^+^ storage behavior in perfect A/C‐V_2_O_3_@C‐HCS and engineered defects‐rich heterophase A/C‐V_2_O_3−_ *_x_* @C‐HMCS. **Figure** **4**a illustrates the atomic model of perfect heterophase system A/C‐V_2_O_3_@C‐HCS (defects free), which consists of a crystalline V_2_O_3_ phase (left) with an amorphous V_2_O_3_ phase (right) obtained via “melt‐and‐quench” method (see details in method section). The crystalline V_2_O_3_ is well‐ordered and contains six‐coordinated vanadium octahedra and four‐coordinated oxygen tetrahedrons, which is verified by the radial distribution function calculations (Figure 4c). Compared with the crystalline phase, the radial distribution functions *g*(*r*) of V and O show broader peaks with V—O distances around 2.0 Å, indicating its distorted local structure (Figure 4c,d). Also, the coordination numbers of V and O exhibit increasing trends, but less than six and four, respectively (Figure 4a,d).

Further, the unsaturated coordinated V and O in the amorphous V_2_O_3_ structure offer various defective sites with energy bands close to the Fermi level. On the other hand, Figure 4b illustrates the defective heterophase system A/C‐V_2_O_3−_ *_x_* @C‐HMCS, consisting of vanadium vacancy crystalline V_2_O_3_ phase (C‐V_2−_ *_x_* O_3_) and rich antisites defect amorphous V_2_O_3−_ *_x_* phase (A‐V_2_O_3−_ *_x_*) including abundant porous channels and oxygen vacancies. The electronic structure of crystalline V_2_O_3_ (C‐V_2_O_3_), amorphous V_2_O_3_ (A‐V_2_O_3_), heterophase V_2_O_3_ (A/C‐V_2_O_3_@C‐HCS), and defective V_2_O_3−_ *_x_* heterophase (A/C‐V_2_O_3−_ *_x_* @C‐HMCS) are revealed by the calculated total density of states (DOSs) (Figure 4e). Distinctly, the bandgap of A‐V_2_O_3_ (0.16 eV) is much narrower than that of C‐V_2_O_3_ (0.49 eV), indicating its high electron mobility and shortened Na^+^ diffusion path.^[^ ^34^, ^46^ ^]^ Further, the A/C‐V_2_O_3_@C‐HCS perfect heterointerface possesses larger DOSs at the Fermi level, higher conductivity, and narrow bandgap (0.12 eV) than its single crystalline V_2_O_3_ and amorphous V_2_O_3_ counterparts, indicating that heterophase is beneficial for Na^+^ storage. In comparison, the A/C‐V_2_O_3−_ *_x_* @C‐HMCS with heterophase and multiple defects exhibits the smallest bandgap (0.06 eV) among all samples, indicating that the multiple defects effectively modulate the electronic distribution and thus account for the shift of conduction band of A/C‐V_2_O_3−_ *_x_* @C‐HMCS toward the Fermi level. The adsorption energies of Na ions (E_ads_) in A/C‐V_2_O_3_@C‐HCS and A/C‐V_2_O_3−_ *_x_* @C‐HMCS were also calculated for comparison (Figure 4f). The A/C‐V_2_O_3_@C‐HCS structure shows a dramatical difference in Na^+^ adsorption kinetics between the crystalline (−0.77 eV) and amorphous (−2.81 eV) phases, which significantly reduces the adsorption energy of Na^+^ at the heterointerface sites (−1.77 eV), reveals that the sparse interfacial sites limit the optimization of adsorption energies, thus hindering the Na^+^ kinetics in A/C‐V_2_O_3_@C‐HCS. In contrast, the defective A/C‐V_2_O_3−_ *_x_* @C‐HMCS heterophase system exhibits lower Na^+^ adsorption energy in either the crystalline phase (−4.09 eV) or the amorphous phase (−4.43 eV), indicating that the defects induce high‐density A/C heterointerface sites with strong synergistic effects. Which can not only modulate the electronic structure, and enrich active sites, but also optimizes adsorption energies (−4.77 eV) at the heterointerfaces sites, thus accelerating Na^+^ kinetics in A/C‐V_2_O_3−_ *_x_* @C‐HMCS (Table S1, Supporting Information). Consequently, the Na^+^ energy barrier of highly dense A/C‐V_2_O_3−_ *_x_* @C‐HMCS heterointerface sites is much lower than that of the sparse A/C‐V_2_O_3_@C‐HCS interfacial sites for both pathways, elucidating that the presence of oxygen and vanadium vacancies promotes Na^+^ diffusion in A/C‐V_2_O_3−_ *_x_* @C‐HMCS (Figure 4g). This result reveals that the abundant unsaturated coordination sites created by the lattice distortion enlarge porous channels for Na^+^ diffusion in the crystalline phase, thereby optimizing its kinetics to be compatible with the oxygen‐vacancy‐rich amorphous phase. Importantly, this greatly reduces the high contrast of the kinetic properties between the crystalline and amorphous phases and induces the highly dense A/C interfaces with a strong synergistic effect, which is consistent with the aforementioned analysis (Figure 4f), implying its outstanding kinetic behavior. Particularly, Na^+^ adsorption energy in vanadium‐deficient crystalline phase C‐V_2−_ *_x_* O_3_ (−4.09 eV) is much lower than that of C‐V_2_O_3_ (−0.77 eV), A‐V_2_O_3_ (−2.81 eV), and C‐V_2_O_3−_ *_x_* (−3.19 eV) (Figure S17, Supporting Information), resulting in higher adsorbing ability and faster kinetics of Na storage in vanadium deficient crystalline structure. In addition, the E_ads_ of A/C‐V_2_O_3−_ *_x_* @C‐HMCS is even lower (Table S1, Supporting Information), which should be ascribed to the interface effect at the dense heterointerface. This result suggests that tailoring unsaturated coordination sites is an effective strategy to enhance electronic transfer. Further, the optimal structure and multifunctional modifications (that is, vanadium vacancy, oxygen vacancy, mesoporous structure, and large specific surface area) endow the A/C‐V_2_O_3−_ *_x_* @C‐HMCS electrode with enhanced ion and electron transfer ability, as well as improved structural stability.

Based on these DFT calculation observations, the highly dense heterointerface in A/C‐V_2_O_3−_ *_x_* @C‐HMCS can significantly narrow the bandgap, optimize adsorption energy, and reduce the Na^+^ diffusion barrier, which endows it with much improved electrochemical Performance. These advantages imply that oxygen‐deficient amorphous V_2_O_3−_ *_x_* hollow mesoporous core and distorted crystalline V_2_O_3_ shell is a promising cathode material for SIBs, as thereafter verified by experimental investigations.

### 2.3 Electrochemical Performance

**Figure 5:**
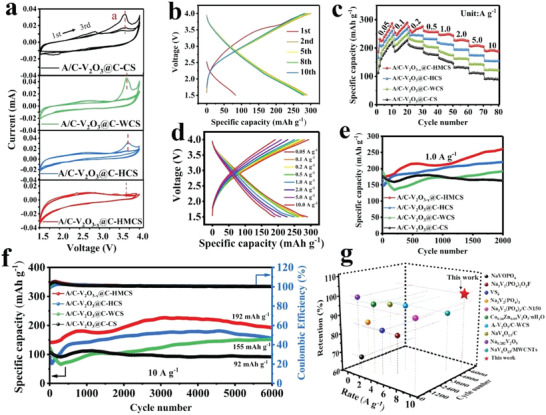
Electrochemical performance of A/C‐V_2_O_3_@C‐CS, A/C‐V_2_O_3_@C‐WCS, A/C‐V_2_O_3_@C‐HCS, and A/C‐V_2_O_3−_ *_x_* @C‐HMCS for sodium‐ion storage. a) CV profiles in the first three cycles at 0.1 mV s^−1^. b) The GCD curves of A/C‐V_2_O_3−_ *_x_* @C‐HMCS at different cycles at 0.1 A g^−1^. c) Rate capability at various current densities. d) Discharge–charge profiles of A/C‐V_2_O_3−_ *_x_* @C‐HMCS at different rates. e) Cycling performance at 1.0 A g^−1^. f) Long‐life cycling performance at 10 A g^−1^. g) Comparison of high rate and long‐term cyclic stability performance with previous vanadium‐based cathodes published works in SIBs.

To verify the feasibility of the high‐density heterointerfaces, the electrochemical performances of a series of A/C‐V_2_O_3_@C‐M‐based cathodes for SIBs were evaluated using the standard coin‐type cell configuration. **Figure** **5**a displays the CV curves at a scan rate of 0.1 mV s^−1^ in the potential range of 1.5–4.0 V. During the first cycle, the A/C‐V_2_O_3_@C‐CS reveals an abroad cathodic peak at ≈2.0 V, corresponding to sodium‐ion insertion, and a sharp anodic peak at ≈3.6 V, assigned to sodium‐ion extraction. In the first anodic scan of A/C‐V_2_O_3_@C‐CS, the peak at 3.6 V is slightly shifted toward a higher voltage in A/C‐V_2_O_3_@C‐WCS and A/C‐V_2_O_3_@C‐HC due to the Faradaic process.^[^ ^20^, ^47^ ^]^ However, the peaks completely disappeared in the subsequent cycles, which is consistent with A/C‐V_2_O_3−_ *_x_* @C‐HMCS CV curves, indicating that the pseudocapacitive dominates the structure.^[^ ^48^ ^]^ Figure 5b exhibits the galvanostatic profiles of the A/C‐V_2_O_3−_ *_x_* @C‐HMCS cathode with different cycles at 0.1 A g^−1^. No obvious plateau was observed in the charge/discharge curves of A/C‐V_2_O_3−_ *_x_* @C‐HMCS at different cycles. The A/C‐V_2_O_3−_ *_x_* @C‐HMCS delivers an initial discharge/charge capacity of 83 and 290 mAh g^−1^. The low initial discharge capacity is corresponding to the insertion of 0.5 Na into the V_2_O_3_ structure (Equation 1). The capacity loss in the first discharge cycle can be assigned to the activation process. After the activation, the half‐cell restores a high discharge capacity of 290 mAh g^−1^ (about 1.6 Na) at the 2nd cycle which is quite close to the theoretical capacity of intercalated 2 Li (i.e. 356 mA h g^−1^) when the oxidation state of V_2_O_3_ reduced to +2 (Equation 2).^[^ ^49^ ^]^ Further, A/C‐V_2_O_3−_ *_x_* @C‐HMCS displays a negligible change in the following cycles, confirming its excellent reversibility.

The reaction occurring of A/C‐V_2_O_3−_ *_x_* @C‐HMCS can be represented by the following equations

(1) $$V_{2}O_{3}+xe+x{\mathrm{Na}}^{+}↔{\mathrm{Na}}_{x}V_{2}O_{3}\left(0<x<0.5\right)$$

(2) $$V_{2}O_{3}+xe+x{\mathrm{Na}}^{+}↔{\mathrm{Na}}_{x}V_{2}O_{3}\left(0<x<2.0\right)$$

Further, as shown in Figure S18a–c (Supporting Information), the A/C‐V_2_O_3−_ *_x_* @C‐HMCS achieves a higher initial capacity (290 mAh g^−1^) than those of A/C‐V_2_O_3_@C‐HCS (268 mAh g^−1^), A/C‐V_2_O_3_@C‐WCS (248 mAh g^−1^), and A/C‐V_2_O_3_@C‐CS (227 mAh g^−1^) cathodes. The rate performances of electrodes were tested under the current density from 0.1 to 10 A g^−1^ (Figure 5c). The A/C‐V_2_O_3−_ *_x_* @C‐HMCS delivers superior reversible capacities of 290, 278, 270, 262, 248, 228, and 208 mAh g^−1^ at a current density of 0.05, 0.1, 0.2, 0.5, 1.0, 2.0, and 5.0 A g^−1^. More importantly, even at an ultrahigh rate of 10 A g^−1^, it still can realize a reversible capacity as high as 192 mAh g^−1^, which is much higher than those of A/C‐V_2_O_3_@C‐HCS (155 mAh g^−1^), A/C‐V_2_O_3_@C‐WCS (123 mAh g^−1^), A/C‐V_2_O_3_@C‐CS (92 mAh g^−1^), as well as most reported vanadium‐based cathode for SIBs (Table S2, Supporting Information). Moreover, the GCD curves of A/C‐V_2_O_3−_ *_x_* @C‐HMCS at various rates display a small polarization, confirming its excellent ion/electron dynamics (Figure 5d). The cycling performance of A/C‐V_2_O_3_@C‐CS, A/C‐V_2_O_3_@C‐WCS, A/C‐V_2_O_3_@C‐HCS, and A/C‐V_2_O_3−_ *_x_* @C‐HMCS samples at a rate of 1.0 A g^−1^ are illustrated in Figure 5e. The A/C‐V_2_O_3_@C‐CS electrode exhibits a high initial capacity of 176 mAh g^−1^, which is fully obtained after 500 cycles and still could retain 93% capacity retention over 2000 cycles. As for comparisons, the A/C‐V_2_O_3_@C‐WCS displays an initial capacity of 191 mAh g^−1^, which is slightly higher than those of A/C‐V_2_O_3_@C‐HCS (181 mAh g^−1^), and A/C‐V_2_O_3−_ *_x_* @C‐HMCS (176 mAh g^−1^), respectively. However, their capacities show obvious degradation over only 250 cycles. After the preceding activated process (500 cycles), the capacities of A/C‐V_2_O_3_@C‐WCS, A/C‐V_2_O_3_@C‐HCS, and A/C‐V_2_O_3−_ *_x_* @C‐HMCS cathodes gradually increase and reach the maximum values of 192, 221, and 261 mAh g^−1^ over 2000 cycles, revealing that all of these heterophases composites show no obvious capacity loss. Notably, an evident capacity rise emerges in the later cycling stage due to the increased surface area of the well‐defined interface, hollow core, and mesoporous structure. Which provides sufficient electrode‐electrolyte contact area and promotes the electrode reaction kinetics, and eventually enhances Na^+^ diffusion on the heterointerfaces.^[^ ^15^, ^50^ ^]^ Obviously, the A/C‐V_2_O_3−_ *_x_* @C‐HMCS exhibits the best cycling performance under high‐rate conditions, achieving excellent kinetic properties at 1.0 A g^−1^. The high‐density heterointerfaces of the A/C‐V_2_O_3−_ *_x_* @C‐HMCS endow it with robust A/C interfacial sites and facilitated ion/electron diffusion, thereby showing superior electrochemical properties to the sparse heterophase interfaces A/C‐V_2_O_3_@C‐CS, A/C‐V_2_O_3_@C‐WCS, A/C‐V_2_O_3_@C‐HCS, which could also be confirmed by the following comprehensive tests.

As the long‐term cycling stability is of critical significance for practical applications, these cathodes were examined at an ultrahigh rate of 10 A g^−1^ (Figure 5f). As expected, the A/C‐V_2_O_3−_ *_x_* @C‐HMCS delivers a decent capacity of 192 mAh g^−1^ over 6000 cycles at a high rate of 10 A g^−1^ with almost 100% capacity retention, as well as coulombic efficiency (Figure 5f), demonstrating outstanding long‐term cycling durability. For both A/C‐V_2_O_3_@C‐WCS and A/C‐V_2_O_3_@C‐HCS cathodes, only a lower capacity of 155 mAh g^−1^ can be provided after 6000 cycles due to their sparse heterointerfaces sites. Meanwhile, the A/C‐V_2_O_3_@C‐CS exhibits an inferior capacity of 92 mAh g^−1^ due to its smaller specific area. These results all identify the highly dense heterointerfaces of A/C‐V_2_O_3−_ *_x_* @C‐HMCS can significantly achieve much more high‐rate and excellent ultralong cyclic stability than sparse heterointerfaces cathodes (A/C‐V_2_O_3_@C‐CS, A/C‐V_2_O_3_@C‐WCS, and A/C‐V_2_O_3_@C‐HCS). Overall, this work highlights that the high dense heterointerface A/C‐V_2_O_3−_ *_x_* @C‐HMCS enables much improved electrochemical performances among vanadium‐based cathodes for SIBs (Figure 5g and Table S2, Supporting Information).^[^ ^33^, ^51^, ^52^, ^53^, ^54^, ^55^, ^56^, ^57^, ^58^, ^59^ ^]^ This splendid stability at an ultrahigh rate is mainly ascribed to the unique construction of nanostructured dense heterointerfaces, including heterophase (A/C), abundant defects, synergistic effects, mesoporous structure, and high surface area that facilitates the electron conduction and ions diffusion, thus boosting the reaction kinetics of A/C‐V_2_O_3−_ *_x_* @C‐HMCS.

### 2.4 Na‐Ion Storage Analysis and Kinetic Study

**Figure 6:**
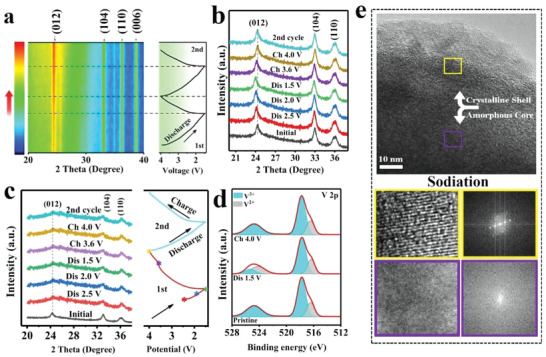
Na‐ion storage process analysis. a) In situ XRD patterns of the A/C‐V_2_O_3_@C‐HCS. The ex situ XRD patterns at various discharged/charged states of b) A/C‐V_2_O_3_@C‐HCS and c) A/C‐V_2_O_3−_ *_x_* @C‐HMCS. d) Ex situ XPS spectra of A/C‐V_2_O_3−_ *_x_* @C‐HMCS at the fully initial de/sodaition process. e) The evolution of the HRTEM image and the I/FFT patterns were taken from the corresponding yellow and violet square areas of the A/C‐V_2_O_3−_ *_x_* @C‐HMCS electrode after 100 cycles.

The phase and structural evolutions of A/C‐V_2_O_3_@C‐HCS and A/C‐V_2_O_3−_ *_x_* @C‐HMCS were monitored by using in/ex situ XRD, Ex‐XPS spectra, and HRTEM to unveil the Na^+^ de/intercalation mechanism (**Figure** **6**). Figure 6a presents the in situ XRD of the A/C‐V_2_O_3_@C‐HCS electrode. In the initial stage, the characteristic peaks of (012), (104), (110), and (006) can be observed at 24.5°,33.1°,36.2°, and 38.5°, respectively. The peak of (012) is slightly negatively shifted, indicating that Na ions have been successfully inserted into V_2_O_3_, as confirmed by ex situ XRD (Figure 6b). On the other hand, no obvious shift in the peaks of A/C‐V_2_O_3−_ *_x_* @C‐HMCS could be observed (Figure 6c), implying a negligible volume change during the Na^+^ insertion/extraction. However, compared to the initial state the intensity of the A/C‐V_2_O_3−_ *_x_* @C‐HMCS peaks is significantly reduced at different stages, which can be attributed to the lattice distortion. The ex situ XPS is carried out to better understand the chemical state of the A/C‐V_2_O_3−_ *_x_* @C‐HMCS during the Na^+^ intercalation/deintercalation mechanism (Figure 6d). In the V 2p XPS region, the reversible electrochemical reduction can be obviously observed due to the insertion of Na^+^. At the fully discharged state (1.5 V), a low valance of V^2+^ is detected in the V 2p_1/2_ peak region, indicating the reduction of V^3+^ to V^2+^ during the Na^+^ insertion into the A/C‐V_2_O_3−_ *_x_* @C‐HMCS (Figure 6d). Yet, the pristine V 2p spectra are well recovered in the fully charged state, which suggests that Na^+^ has been reversibly extracted from the electrode. Moreover, the V^+2^ content is significantly observed even after 100 cycles, indicating an increase in the capacity which is in good agreement with electrochemical performance (Figure 5, Figure S19, Supporting Information). As shown in Figure S20, Supporting Information, there is no Na 1s signal being detected at the initial state but a sharp Na 1s peak appears at a fully discharged state, suggesting the successful insertion of Na^+^. Ex situ TEM measurements are carried out at full sodiation state (1.5 V) to reveal the structural change of A/C‐V_2_O_3−_ *_x_* @C‐HMCS (Figure 6e). From the HRTEM analysis and its corresponding I/FFT patterns, the A/C‐V_2_O_3−_ *_x_* @C‐HMCS is still composed of a clear lattice‐distorted crystalline shell and a defective amorphous core. It is unveiled that the dense A/C interfacial sites can be well maintained even after long‐term cyclability. As shown in the SEM image in Figure S21, Supporting Information, the A/C‐V_2_O_3−_ *_x_* @C‐HMSC still remained in its original morphology even after 1000 cycles, verifying its robust stability during the repeated Na^+^ insertion/extraction processes.

**Figure 7:**
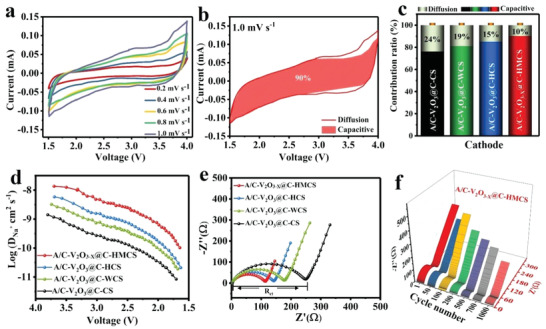
Kinetics and electrochemical reaction behavior of A/C‐V_2_O_3_@C‐M based cathodes. a) CV curves of A/C‐V_2_O_3−_ *_x_* @C‐HMCS electrode at different scan rates and b) its diffusion and capacitive contributions at 1.0 mV s^−1^. Comparison of c) pseudocapacitive contribution of A/C‐V_2_O_3_@C‐CS, A/C‐V_2_O_3_@C‐WCS, A/C‐V_2_O_3_@C‐HCS, and A/C‐V_2_O_3−_ *_x_* @C‐HMCS at 1.0 mV s^−1^, d) Na^+^ diffusion coefficients (D_Na+_) during the sodiation process, and e) Nyquist impedance curves. f) Nyquist plots of A/C‐V_2_O_3−_ *_x_* @C‐HMCS along with different cycles.

To further evaluate the influence of the dense heterophase on the reaction kinetics of A/C‐V_2_O_3−_ *_x_* @C‐HMCS, CV, GITT, and EIS spectra are systematically investigated (**Figure** **7**). Based on this, CV curves were recorded at various scan rates (0.2–1.0 mV s^−1^) (Figure 7a, Figure S22, Supporting Information). The anodic and cathodic peaks are well maintained at different scan rates, suggesting the high reversibility of the heterophase electrodes. The total charge transfer process can be divided into the pseudocapacitive‐controlled process and the diffusion‐controlled process by the following equation:*i* = *av^b^*, where a and b are adjustable parameters.^[^ ^60^, ^61^ ^]^ A b‐value close to 0.5 represents a diffusion‐controlled process, while a b‐value close to 1.0 represents a pseudocapacitive‐controlled process. The diffusion‐controlled charge storage is more sluggish while the pseudocapacitive charge storage is intrinsically faster.^[^ ^62^ ^]^ As shown in Figure S23a,b (Supporting Information), all the b‐values of the A/C‐V_2_O_3−_ *_x_* @C‐HMCS are close to 1.0, implying that the pseudocapacitive dominates the batteries (Figure), ensuring its fast reaction kinetics for excellent rate capability in SIBs (Figure 5c,f). Further, the relative diffusion and capacitive‐controlled process at specific voltage are quantitatively analyzed by the following equation

(3) $$i\left(v\right)=k_{1}v+k_{2}v^{1/2}$$

where *k*_1_ *v* and *k*_2_ *v* ^1/2^ represent reaction and diffusion processes, respectively.^[^ ^48^ ^]^ The capacitive contribution ratio of all electrodes at a scan rate of 1 mV s^−1^ is displayed in Figure 7b,c. Among them, the A/C‐V_2_O_3−_ *_x_* @C‐HMCS electrode realizes the highest capacitive contribution ratio (90%), implying the domination of capacitive control. This ultrahigh capacitive contribution of A/C‐V_2_O_3−_ *_x_* @C‐HMCS is achieved from the sufficient activated Na^+^ sites and enhanced electron conductivity, resulting in superior electrochemical kinetic with extraordinary rate performance.

The reaction resistance and Na‐ion diffusion coefficient D_Na+_ in heterophase electrodes were investigated by electrochemical galvanostatic intermittent titration technique (GITT) and Nyquist impedance spectra. Figure 7d and Figure S24 (Supporting Information) present the GITT curves of electrodes during sodiation and desodiation process, respectively. As expected, the A/C‐V_2_O_3−_ *_x_* @C‐HMCS electrode shows higher D_Na+_ values than those of A/C‐V_2_O_3_@C‐HCS, A/C‐V_2_O_3_@C‐WCS, and A/C‐V_2_O_3_@C‐CS at the entire cycle, which validates its most superior diffusion kinetics among all samples. The EIS spectra and equivalent circuit diagrams are shown in Figure 7e. The semicircle in high frequency and the line in low frequency can be ascribed to the charge transfer resistance *R*_ct_ and the Warburg diffusion process *Z*_w_, respectively. The A/C‐V_2_O_3−_ *_x_* @C‐HMCS displays the lowest *R*_ct_ (102 Ω) than that of A/C‐V_2_O_3_@C‐HCS (137 Ω), A/C‐V_2_O_3_@C‐WCS (165 Ω), and A/C‐V_2_O_3_@C‐CS (245 Ω), which further proves that the synergy of heterophase and defects can effectively reduce charge transfer resistance and accelerate electron transfer. Further, the *R*_ct_ values of the A/C‐V_2_O_3−_ *_x_* @C‐HMCS electrode decrease gradually with increasing cycling number, which contributes to the increase in capacitance.^[^ ^63^ ^]^ Such superior results demonstrate that the vanadium and oxygen vacancies and larger surface area of A/C‐V_2_O_3−_ *_x_* @C‐HMCS could induce more active sites to store Na^+^, and thus benefit the fast kinetics. These results further verify that the highly dense heterointerface core–shell cathode A/C‐V_2_O_3−_ *_x_* @C‐HMCS realizes the balance between high stability and fast kinetics.

## 3 Conclusion

In summary, we report the design of unique engineered defects‐rich heterophase core–shell (A/C‐V_2_O_3−_ *_x_* @C‐HMCS) composed of oxygen‐deficient amorphous V_2_O_3−_ *_x_* hollow mesoporous core (A‐V_2_O_3−_ *_x_*/HMC) and lattice‐distorted crystalline V_2_O_3_ shell (C‐V_2_O_3_/S) encapsulated by carbon, which is successfully prepared via one‐pot hydrothermal method followed by a reduction reaction and activation process. The abundant unsaturated coordination sites created by lattice distortion enlarged Na^+^ diffusion channels in the crystalline phase, thereby optimizing its kinetics to be compatible with the oxygen‐vacancy‐rich amorphous phase. The introduction of defects effectively enriches active sites and modulates the electronic configuration in heterointerface sites. This significantly reduces the high contrast of the kinetic properties between the crystalline and amorphous phases and induces the formation of dense highly dense A/C interfaces, with a strong synergistic effect. In contrast, the perfect heterophase (defects‐free) A/C‐V_2_O_3_@C‐HCS demonstrates sparse interfacial sites with a limited synergistic effect. DFT calculations further reveal that the dense heterointerfaces efficiently optimize Na^+^ adsorption energies, and lower Na^+^ diffusion barriers, thus promoting Na^+^ kinetics. In addition, the ex‐HRTEM and ex‐XRD results indicate that the dense A/C interfacial sites can be well maintained even after long‐term cyclability, revealing the robust stability of A/C‐V_2_O_3−_ *_x_* @C‐HMCS. As a result, such unique A/C‐V_2_O_3−_ *_x_* @C‐HMCS exhibits outstanding electrochemical performances, with a high rate (261 mAh g^−1^ at 1.0 A g^−1^ over 2000 cycles) and ultrastable performance (192 mAh g^−1^ over 6000 cycles at 10 A g^−1^) when first used as a cathode for SIBs, outperforming most of the reported SIBs cathodes. According to our aforementioned experimental evidence, it has been demonstrated that the electrochemical properties of a cathodic material depend not only on the oxidation state of the material but also largely on its crystal structure and morphology. This work provides new fundamental insights into the design of dense heterophase electrodes for electrochemical energy storage and beyond.

## 4 Experimental Section

### Materials Synthesis—Synthesis of A/C‐V_2_O_3_@C‐CS and A/C‐V_2_O_3_@C‐WCS

The V_2_O_5_ spheres precursor (V‐glycerate) was prepared as described in a previous hydrothermal method.^[^ ^33^ ^]^ In a typical procedure, 0.6 mL VO(OiPr)_3_ vanadium isopropoxide was added to a mixture of 6 mL glycerol and 60 mL isopropyl alcohol and magnetically stirred for 15 min. Then, the mixture was transferred into an autoclave and kept at 180 °C for 6 h. After it naturally cooled, the precipitate was centrifuged, washed, and finally dried at 80 °C. Afterward, the precursor was calcined under the Ar/H_2_ (95:5%) atmosphere for 2 h at 500 °C, and the carbon‐coated amorphous/crystalline V_2_O_3_ heterophase core–shell was achieved (donated as A/C‐V_2_O_3_@C‐CS). Further, the well‐defined heterophase A/C‐V2O3@C‐WCS was prepared by calcining of A/C‐V_2_O_3_@C‐CS for 2 h at 500 °C under N_2_.

### Materials Synthesis—Synthesis of A/C‐V_2_O_3_@C‐HCS and A/C‐V_2_O_3−_ *_x_* @C‐HMCS

For comparison, the A/C‐V_2_O_3_@C‐HCS was also prepared under the same reaction condition as the synthesis of the A/C‐V_2_O_3_@C‐CS, except 1.0 mL of distilled water was dropwise added to the mixture to obtain hydrated V‐glycerate precursor. Then, the precursor was calcined under the Ar/H_2_ (95:5%) atmosphere for 2 h at 600 °C to achieve carbon‐coated V_2_O_3_ heterophase hollow core–shell A/C‐V_2_O_3_@C‐HCS. The carbon‐coated detective‐rich heterophase hollow core–shell A/C‐V_2_O_3−_ *_x_* @C‐HMCS was obtained by calcining of A/C‐V_2_O_3_@C‐HCS under N_2_ for 2 h at 600 °C.

### Characterization

The phase structure of as‐prepared samples was characterized by XRD measurements (Rigaku smart Lab with Cu K*α* radiation, *λ* = 0.15405 nm). The surface state analysis was conducted on X‐ray photoelectron spectroscopy (XPS, UlvacPhi). The morphology and microstructure of the samples were observed by a field emission transmission electron microscope TEM (FEI Talos F200X) and field emission SEM (JSM7500F‐ 5KV and Phenom XL‐15 kV JEOL). The defects were identified by Electron paramagnetic resonance (EPR, Bruker EMXPLUS). The carbon content was analyzed by TGA (40–700 °C with a rate of 5 °C min^−1^ under a pure O_2_ atmosphere)

### Electrochemical Measurements

The working electrode was prepared by stirring 70 wt% active material, 20 wt% carbon black, and 10 wt% polyvinylidene fluoride (PVDF) in N‐methyl pyrrolidone. The obtained slurry was coated on Al foil and vacuum dried at 80 °C. Next, 1 m of NaClO4 in EC: PC = 1:1 with 5% FEC, Whatman glass fiber, and sodium metal were used as the electrolyte, separator, and anode, respectively. CR2032 coin cells were assembled in an Ar‐filled glove box (O_2_ < 0.1 ppm and H_2_O < 0.1 ppm). The mass loading of active material on the electrode was about 1.5 mg cm^−2^. The electrochemical properties of the cells including rate performance, galvanostatic charge/discharge (GCD), and GITT were all tested on the LAND CT2001A system within a voltage range from 1.5 to 4.0 V (vs Na^+^/Na) at room temperature. The CV measurements at different scan rates and EIS spectra were conducted on Gamry‐Reference 1000 electrochemical workstation.

### Computational Method

All DFT calculations were carried out by using the Vienna ab initio simulation package (VASP).^[^ ^64^ ^]^ The valence electrons and ionic core interaction were described by the plane‐wave basis sets and projected augmented wave (PAW) method.^[^ ^65^ ^]^ The geometry optimizations have been performed onto A Г k‐mesh, 450 eV cut‐off kinetic energy, and 0.03 eV Å^−1^ force convergence criterion. The hybrid crystalline/amorphous V_2_O_3_ heterophase was constructed by the “melt‐and‐quench” method and ab initio molecular dynamics (AIMD), in which half of the supercells were fixed and half of the crystalline V_2_O_3_ supercells were melting at a high temperature of 4000 K and cooling down to 300 K in the NVT integrated framework to obtain a crystalline/amorphous V2O3 heterophase structure. For the AIMD simulation, a 2 fs time step was selected with a total simulation time of 18 ps. The 3d orbitals of V were corrected by the Hubbard U term with *U*_eff_ = 3.25 eV. The weak dispersion effect was considered by the DFT‐D3 method.^[^ ^66^, ^67^ ^]^

## Conflict of Interest

The authors declare no conflict of interest.

## Supporting information

Supporting Information

## Data Availability

The data that support the findings of this study are available from the corresponding author upon reasonable request.
