# Supplementary material for: Defect‐Induced Dense Amorphous/Crystalline Heterophase Enables High‐Rate and Ultrastable Sodium Storage
Source: Adv Sci (Weinh). 2022 Oct 30;9(36):2205575. doi: 10.1002/advs.202205575 (PMC9798978; doi:10.1002/advs.202205575)
Supplement: Supplementary file 1 — Supporting Information [file ADVS-9-2205575-s001.pdf]

Supporting Information

Defect-Induced Dense Amorphous/Crystalline Heterophase Enables High-Rate and Ultra-Stable Sodium Storage

Sahar Osman, Chao Peng*, Fangkun Li, Haoliang Chen, Jiadong Shen, Zeming Zhong, Wenjie Huang, Dongfeng Xue, and Jun Liu*

S. Osman, F. Li, H. Chen, J. Shen, Z. Zhong, W. Huang, Prof. J. Liu

School of Materials Science and Engineering and Guangdong Provincial

Key Laboratory of Advanced Energy Storage Materials

South China University of Technology

Guangzhou, Guangdong 510641, China

Email: msjliu@scut.edu.cn

Dr. C. Peng, Prof. D. Xue

Multiscale Crystal Materials Research Center

Shenzhen Institute of Advanced Technology

Chinese Academy of Science, Shenzhen 518055, China

Email: chao.peng@siat.ac.cn

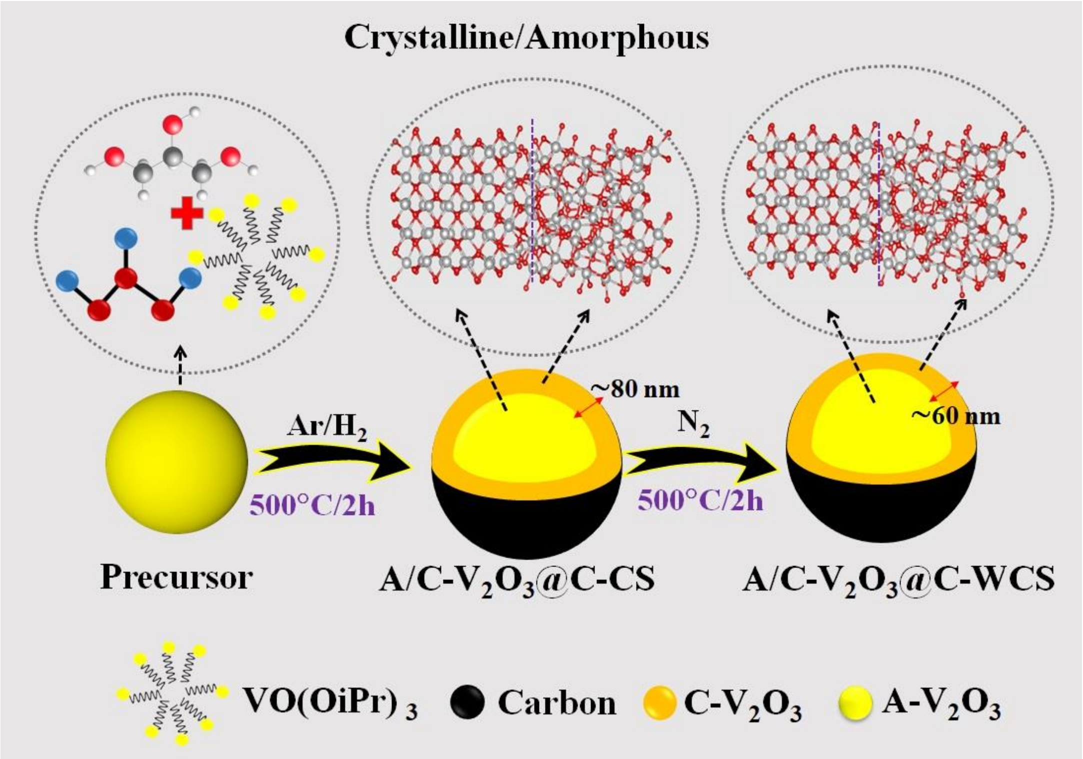

Figure S1. Schematic illustration of the synthesis of A/C-V_2O_3@C-CS and A/C-V_2O_3@C-WCS.

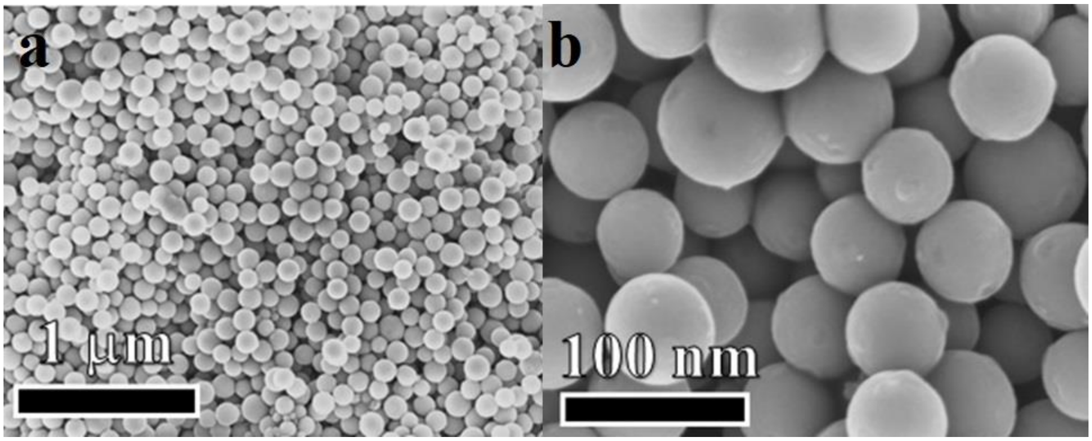

Figure S2. (a, b) Low/high magnification SEM images of vanadium-glycerate precursor.

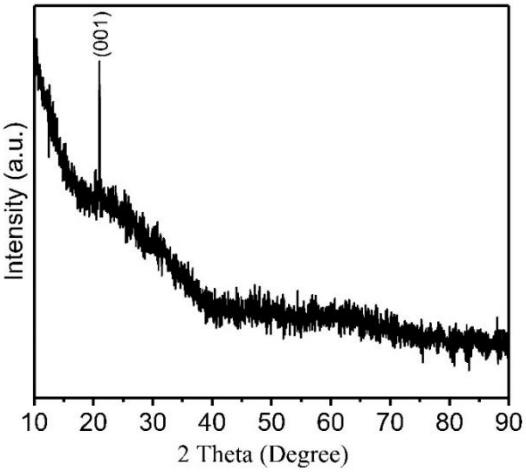

Figure S3. XRD pattern of V-glycerate spheres.

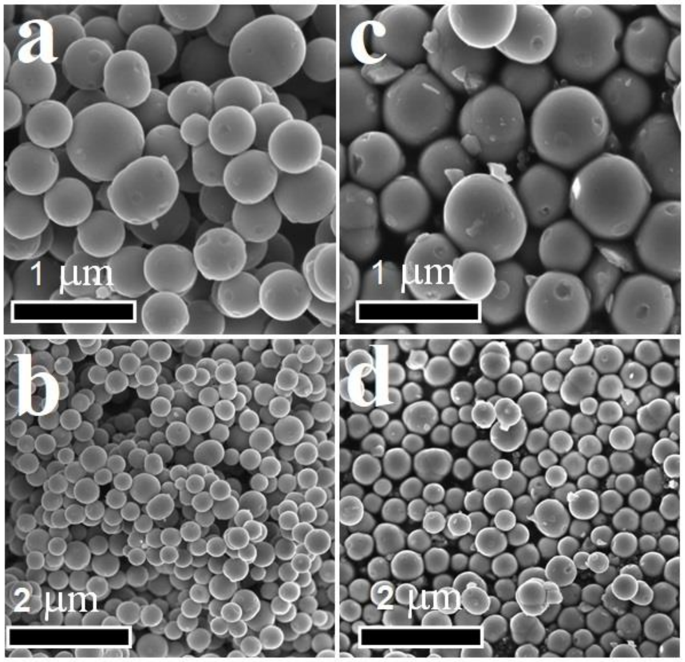

Figure S4. High/low-magnification SEM images of (a, b) A/C-V_2O_3@C-CS and (c, d) A/C-V_2O_3@C-WCS.

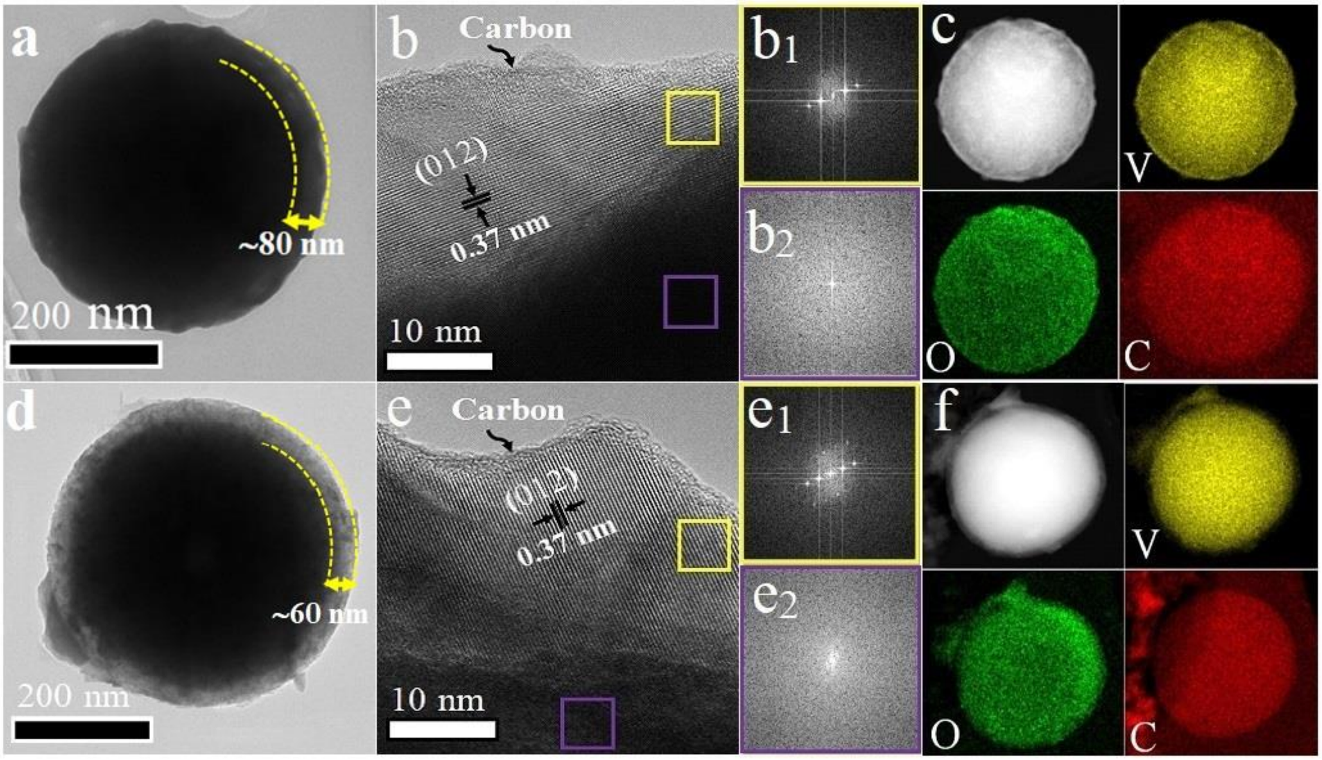

Figure S5. Morphology and characterizations of A/C-V_2O_3@C-CS and /C-V_2O_3@C-WCS (a, d) TEM, (b, e) HRTEM images. (b_1, e_1) FFT patterns are taken from the corresponding yellow square and (b_2, e_2) violet square areas in (b, e), and (c, f) EDS mapping images of core-shell A/C-V_2O_3@C-CS and A/C-V_2O_3@C-WCS, respectively.

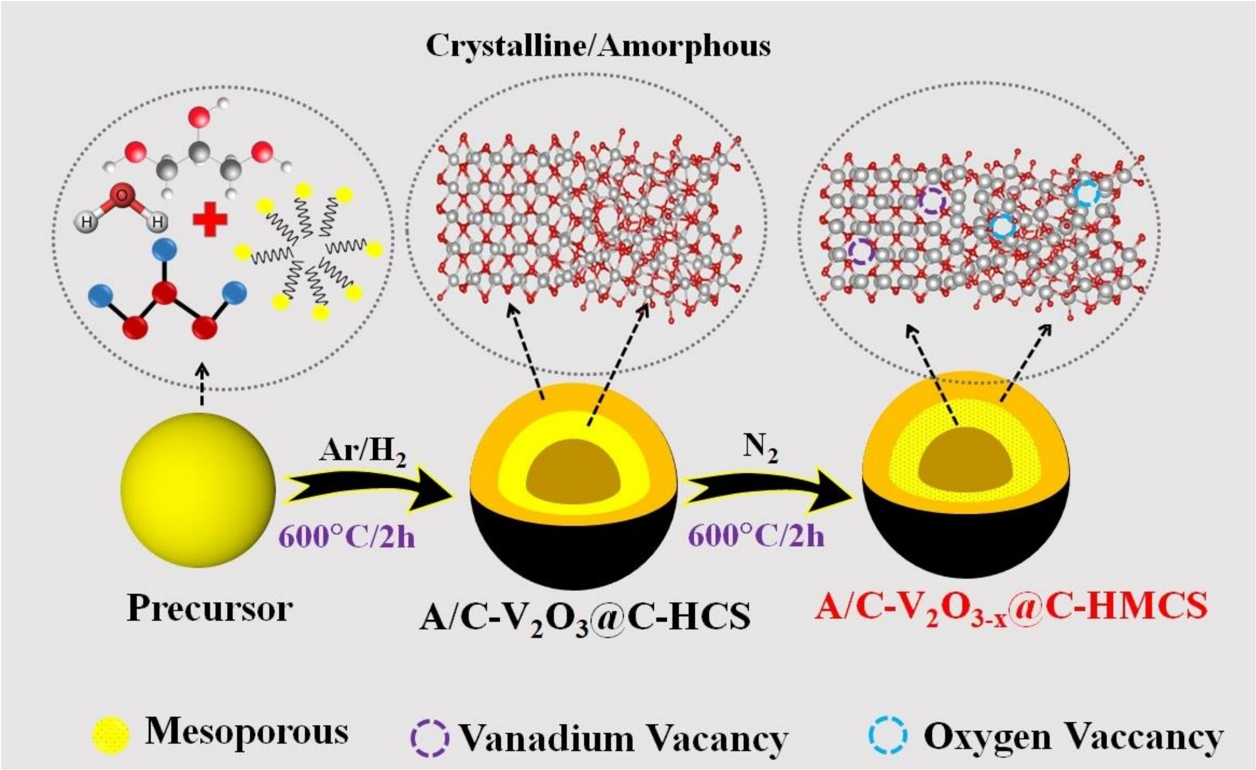

Figure S6. Schematic illustration of the synthesis of A/C-V_2O_3@C-HCS and A/C-V_2O_{3-x}@C-HMCS.

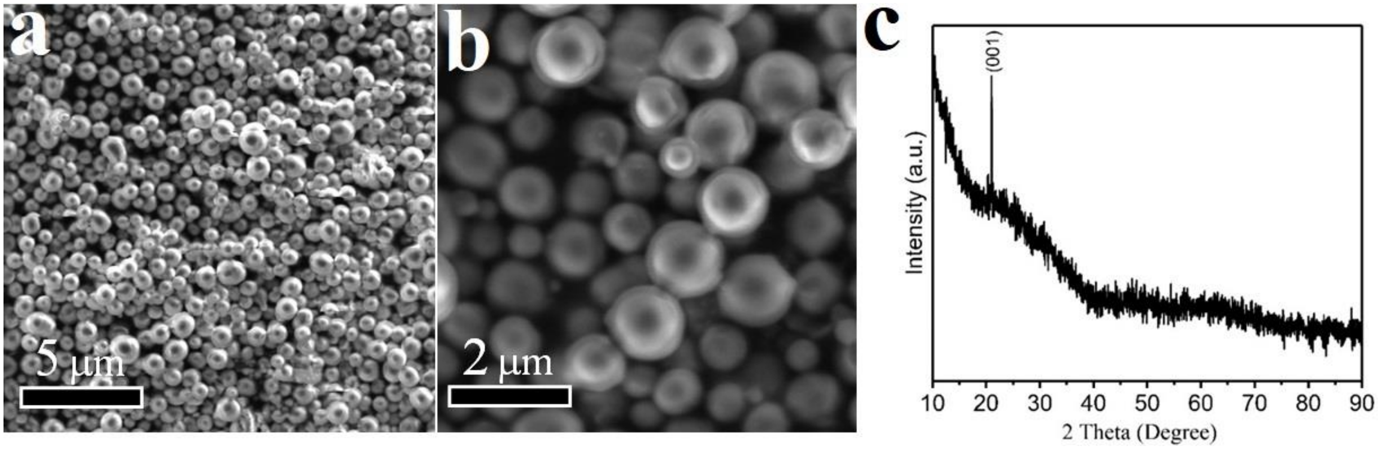

Figure S7. (a, b) Low/high magnification SEM images of vanadium hydrate-glycerate precursor. c) its XRD pattern.

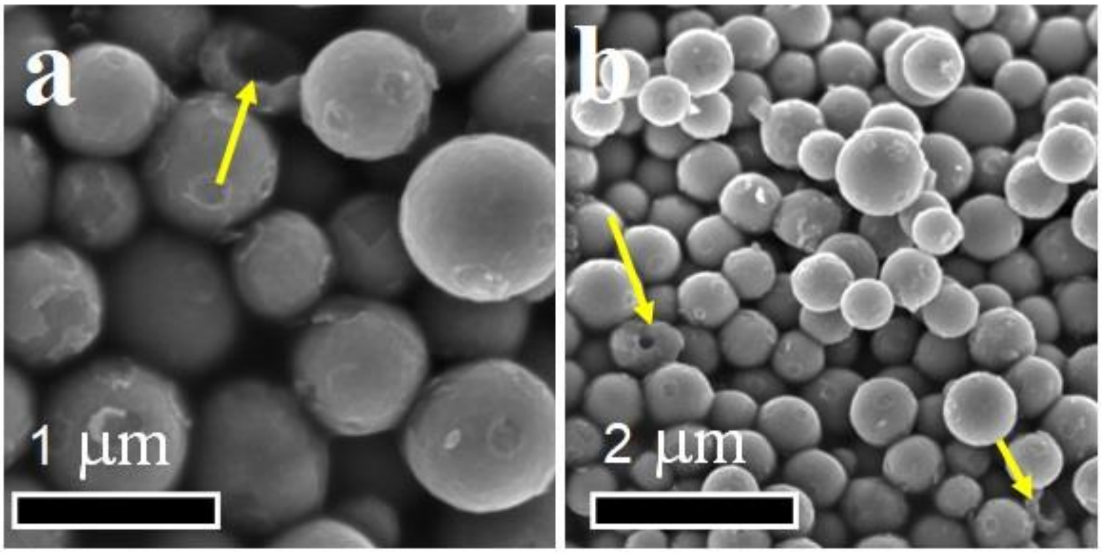

Figure S8. (a, b) High/low-magnification SEM images of A/C-V_2O_3@C-HCS.

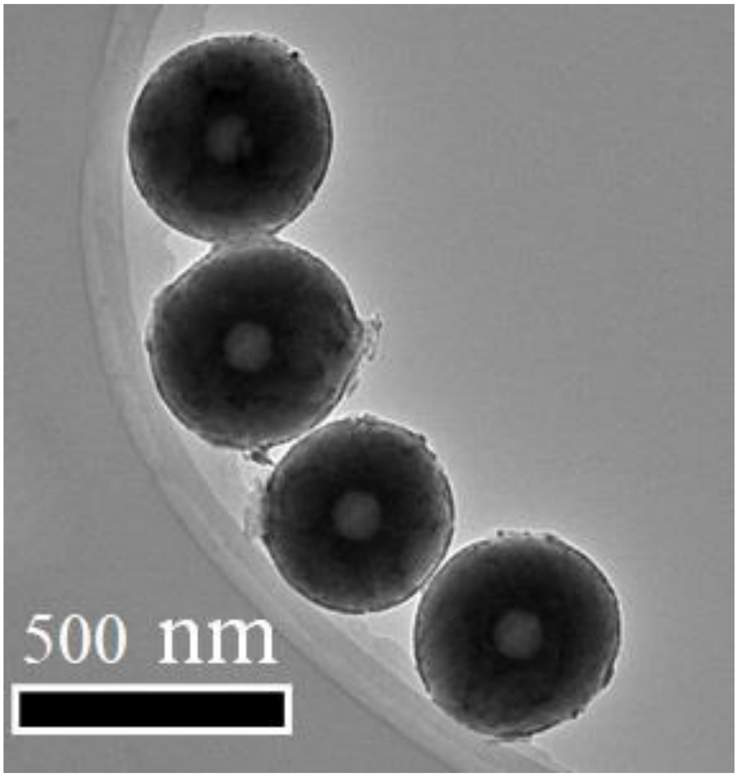

Figure S9. TEM image of A/C-V_2O_3@C-HCS.

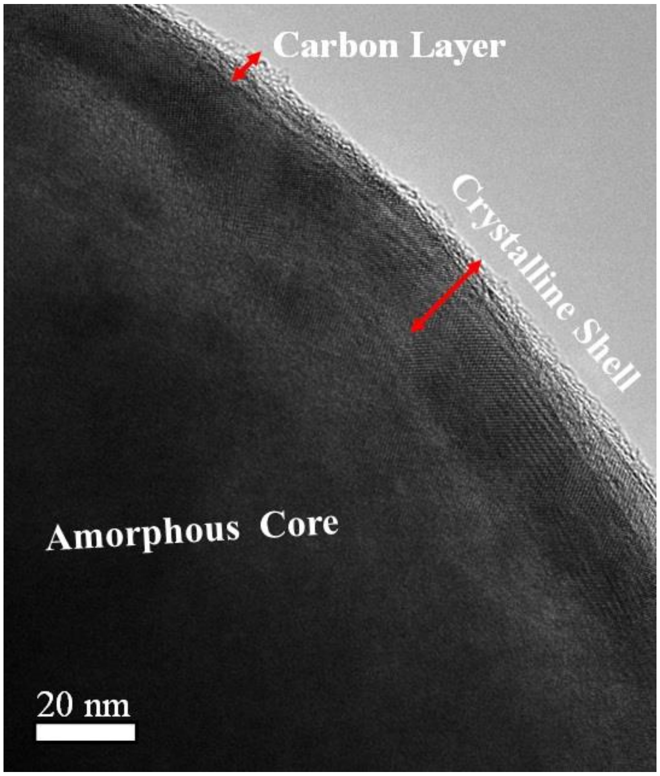

Figure S10. HTEM image of A/C-V_2O_3@C-HCS.

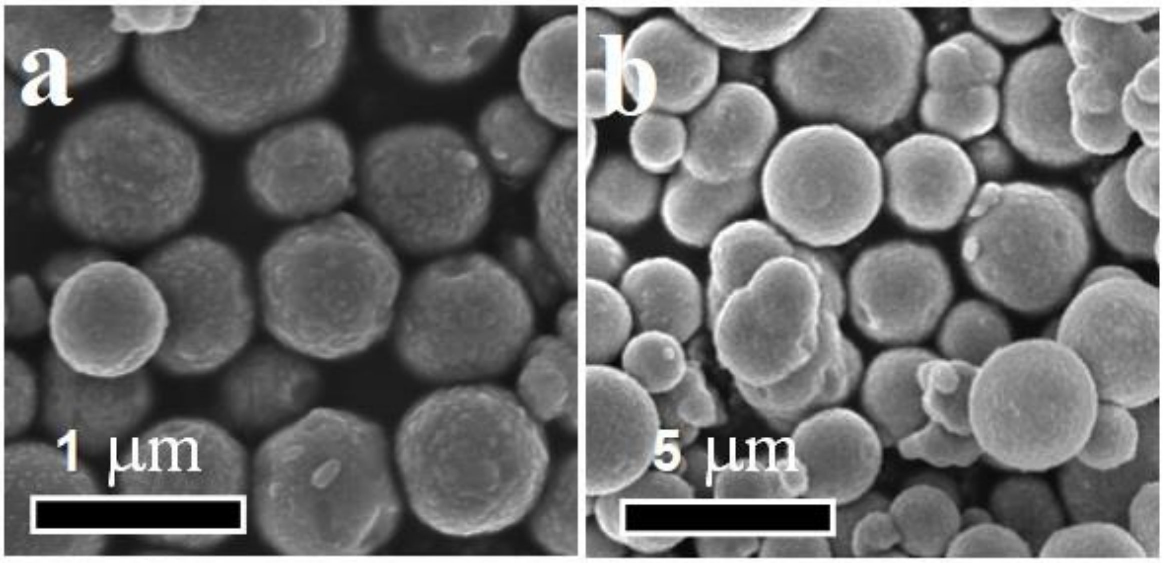

Figure S11. (a, b) High/low-magnification SEM images of A/C-V_2O_{3-x}@C-HMCS.

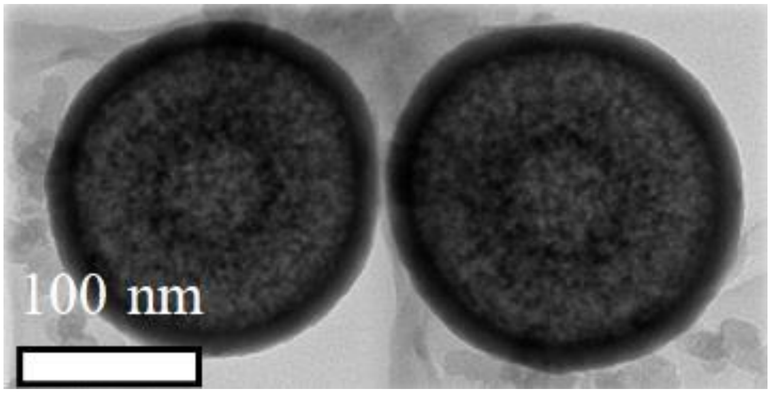

Figure S12. TEM image of A/C-V_2O_{3-x}@C-HMCS.

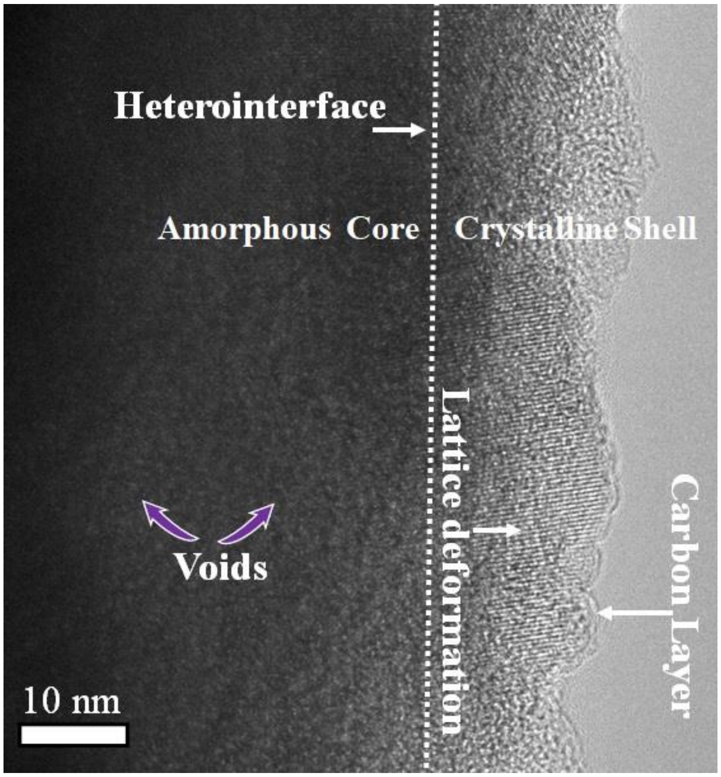

Figure S13. HTEM image of A/C-V_2O_{3-x}@C-HMCS.

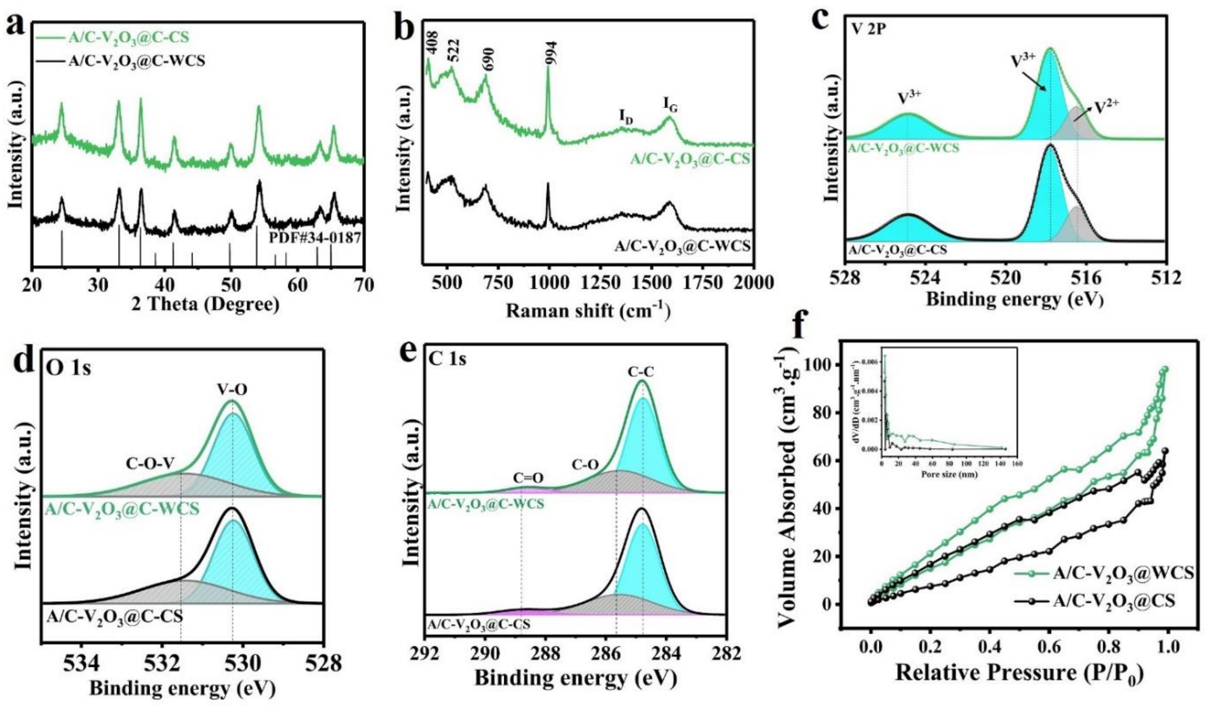

Figure S14. Structural characterizations of A/C-V_2O_3@C-CS and A/C-V_2O_3@C-WCS: (a) XRD patterns, (b) Raman spectra, high-resolution spectra of :(c) V 2p, (d) O 1s, (e) C 1s. (f) N_2 adsorption-desorption isotherms and their pore size distributions (inset).

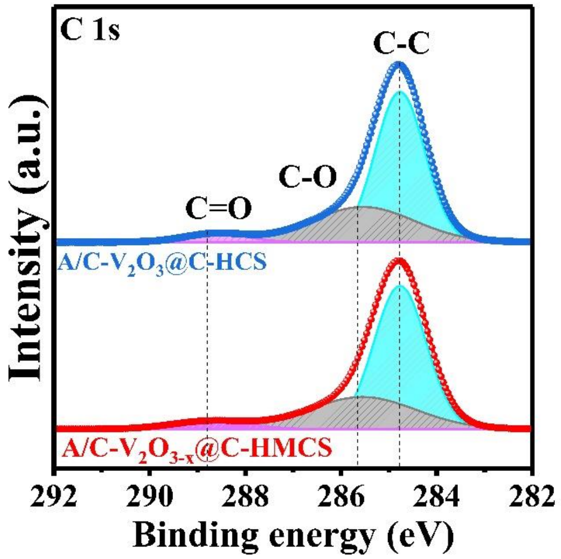

Figure S15. C 1s high-resolution spectra of A/C-V_2O_3@C-HCS and A/C-V_2O_{3-x}@C-HMCS.

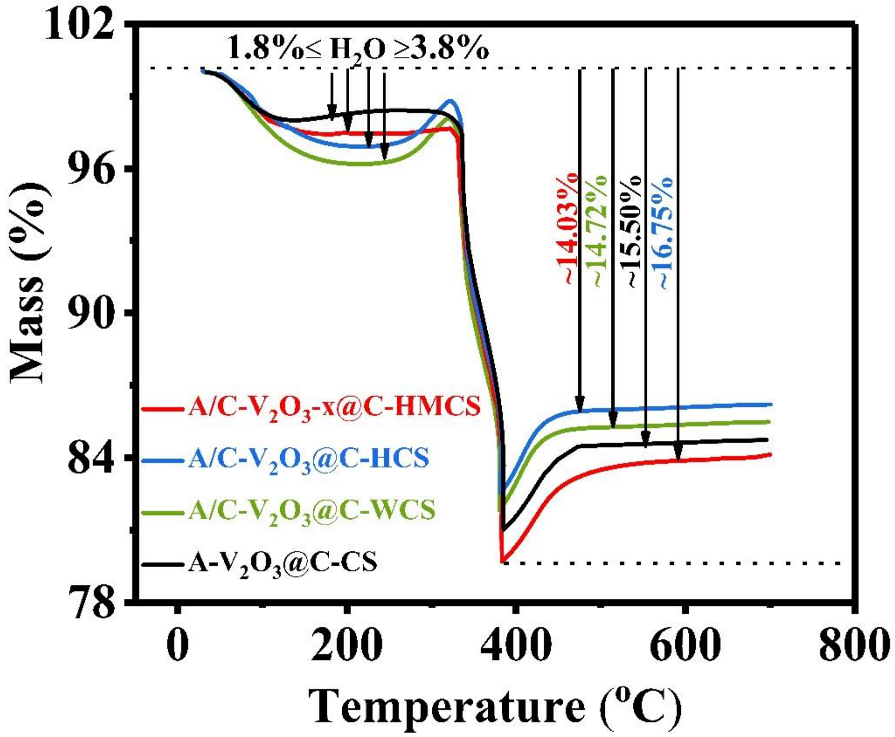

Figure S16. Thermogravimetric analysis (TGA) curve for the A/C-V_2O_3@C-CS, A/C-V_2O_3@C-WCS, A/C-V_2O_3@C-HCS, and A/C-V_2O_{3-x}@C-HMCS.

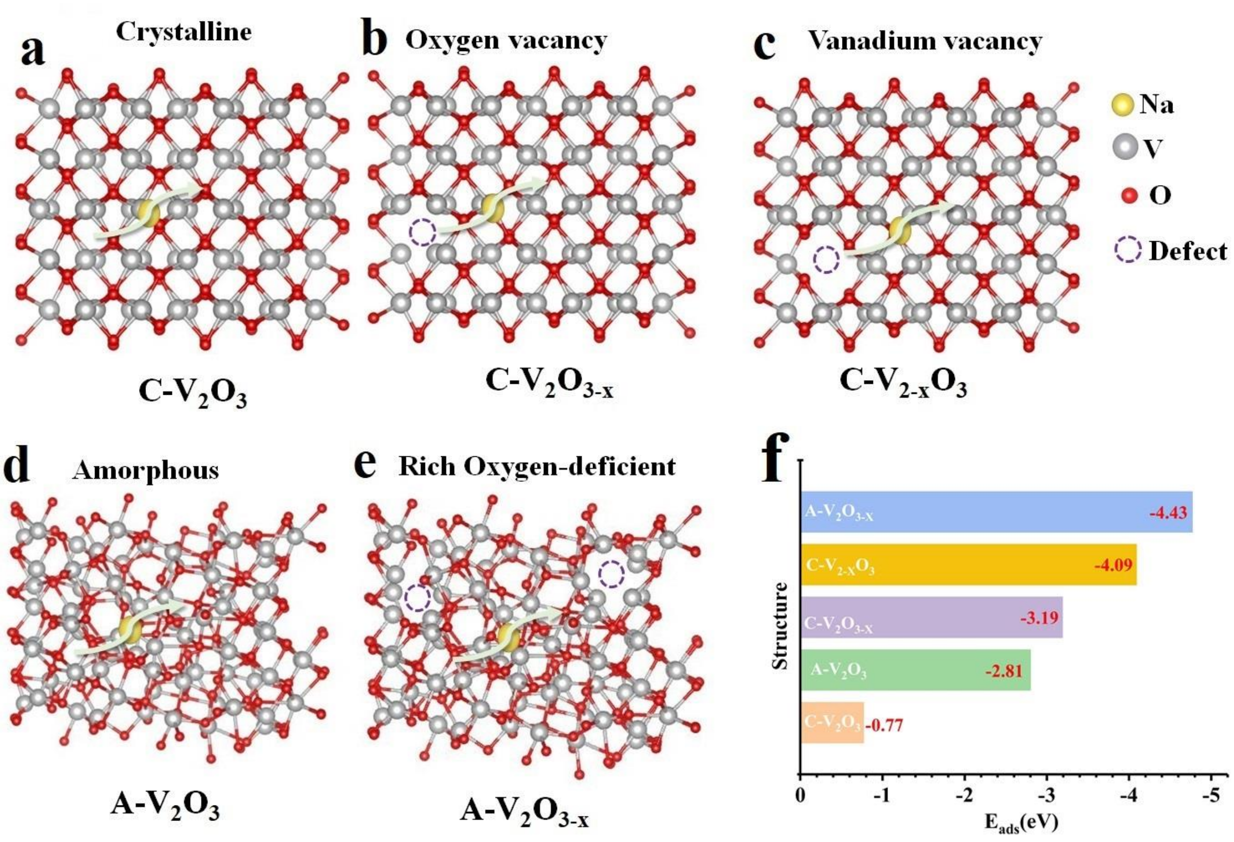

Figure S17. Structure models of Na diffusion in (a) C-V_2O_3 , (b) C-V_2O_{3-x} , (c) C-$V_{2-x}O_3$, (d) A-V_2O_3 ,(e) A-V_2O_3, and (f) their corresponding Na adsorption energies).

Supplemental Table S1: Adsorption energy of Na in various V_2O_3 structures.

**Structure**	**E_{ads} (eV)**
C-V_2O_3	-0.77
A-V_2O_3	-2.81
C-V_2O_{3-x}	-3.19
C-$V_{2-x}O_3$	-4.09
A-V_2O_{3-x}	-4.43
A/C-V_2O_3@C-HMCS	-1.77
A/C-V_2O_{3-x}@C-HMCS	-4.77

Theorectical discussion of Na in various V_2O_3 structures

The adsorption energy (E_{ads}) of the Na atom at amorphous structure is calculated to be -2.81 eV which is much lower than that of crystalline structure (-0.77 eV), indicating that porous isotropic amorphous phase is favorable for Na intercalation (Figure S17a,d). In addition, the Eads of C-V_2O_{3-x} is even lower -3.19 eV (Figure S17b), which should be ascribed to oxygen vacancies, suggesting that the O-defective is highly beneficial for Na insertion. Distinctly,vanadium-deficient crystalline structure C-$V_{2-x}O_3$ displays lower Eads -4.09 eV than crystalline, amorphous, and O-deficient crystalline structure, resulting in higher adsorbing ability and faster kinetics of Na storage (Figure S17c). This enhancement is attributed to a higher coordination number and radius of the vanadium atom compared to the oxygen atom, which leads to enlarged Na^+ diffusion channels in the crystalline phase, thereby optimizing its kinetics. Further by combining the porous amorphous structure and oxygen-deficient, the A-V_2O_{3-x} possesses a much lower Na^+ adsorption energy of -4.43 eV compared to all single structures counterparts (Figure S17e), suggesting A-V_2O_{3-x} is the most energetically favorable for Na insertion. This result suggests that tailoring unsaturated coordination sites is an effective strategy to enhance the adsorption of Na^+ (Figure S17f, Table S1).

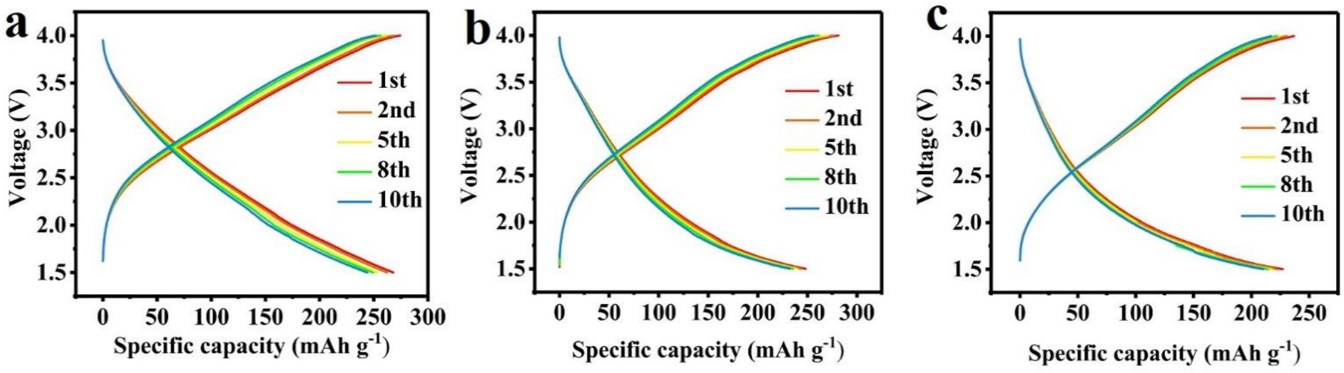

Figure S18. Discharge/charge profiles of (a) A/C-V_2O_3@C-HCS, (b) A/C-V_2O_3@C-WCS, and (c) A/C-V_2O_3@C-CS electrodes at 0.1 A g^{-1}.

Supplemental Table 2: Comparison of the electrochemical performance with reported A/C-V2O3@C-M based electrodes.

Cathode description	Capacity Retention (%, cycles No)	Rate performance (mAh g^{-1})/ (A g^{-1})	Average voltage (V)	Ref
A/C-V_2O_3@C-CS core-shell	93%,6000	92/10	2.6	This work
A/C-V_2O_3@C-WCS core-shell	100%,6000	123/10	2.7	
A/C-V_2O_3@C-HCS core-shell	100%,6000	155/10	2.8	
A/C-V_2O_{3-X}@C-HMCS core-shell	100%,6000	192/10	2.8	
NaV_6O_{15} /MWCNTs nanotube	96%,500	123.7/10	2.7	[1]
A-V_2O_5/C-WCS core-shell	95%,3000	148/5.0	3.0	[2]
Bilayered V_2O_5	85%,320	150/0.63	3.0	[3]
Additive-free V_2O_5 thin film	100%,200	124/5.0	-0.25	[4]
$Co_{0.16}Zn_{0.09}V_2O_5\cdot nH_2O$	97%,1000	90/3.0	0.7	[5]
cG/VO microspheres	73%,2000	214/4.0	1.0	[6]
VS_4 Nanoparticles	84%,1200	188.1/4.0	1.2	[7]
NaV_6O_{15} nanotube	94%,3000	105/2.5	2.5	[8]
$NaVOPO_4$ layered	67%, 1000	57/0.73	3.3	[9]
$Na_{0.282}V_2O_5$ nanorods	99%,1000	80/0.3	2.7	[10]
$Na_{1.25}V_3O_8$ nanowires	87%,1000	76/2.0	2.4	[11]
$Na_3V_2(PO_4)_3$ spheres	87.5%,1000	96/2.0	3.35	[12]
$Na_3V_2(PO_4)_2O_2F$ nanotetraprisms	81%,2000	84/5.2	3.8	[13]
$Na_3V_2(PO_4)_2O_2F$ carbon cloth	95%,1500	84/1.2	3.8	[14]
$Na_3V_2(PO_4)_2F_3$ microcubes	98%,2000	53/2.2	3.6	[15]
$Na_6Fe_5(SO_4)_8$/CNTs nano-architectured	100.8%,1000	86.4/ 0.24	3.6	[16]

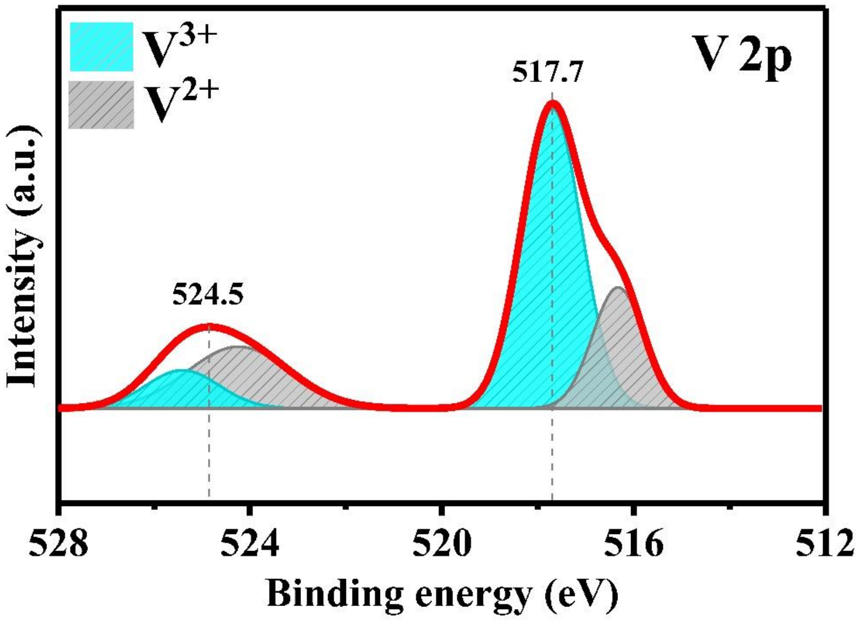

Figure S19. XPS spectrum of V 2p with 100 cycles for A/C-V_2O_{3-x}@C-HMCS electrode.

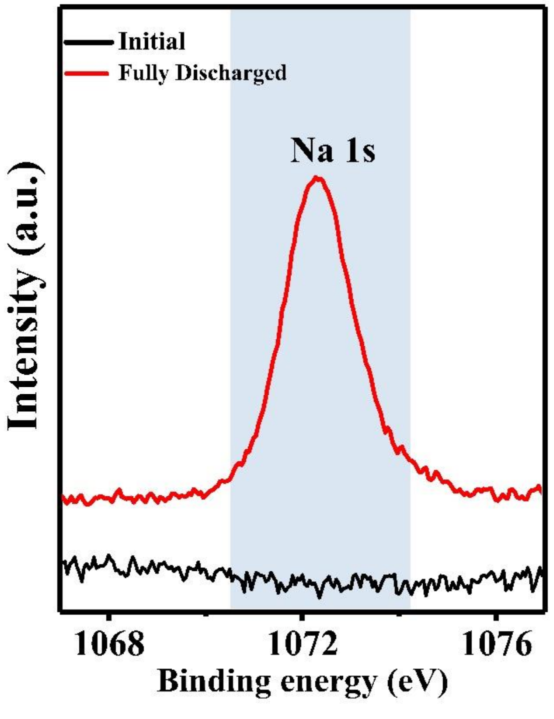

Figure S20. Ex-situ XPS Na 1st spectra of A/C-V_2O_{3-x}@C-HMCS electrodes at full discharge–stage (1.5 V).

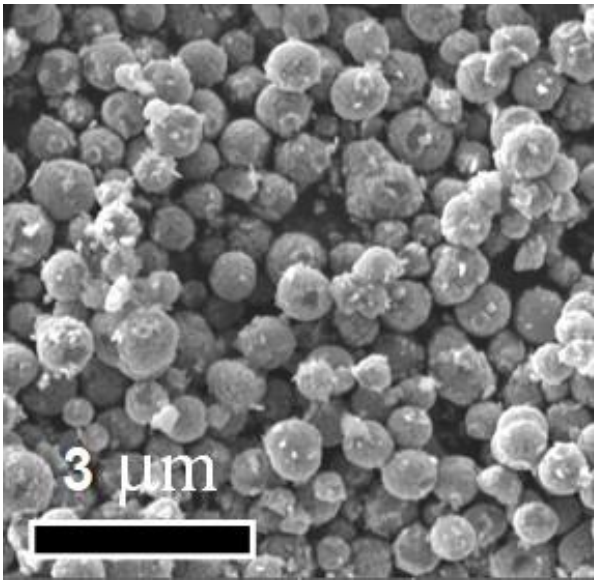

Figure S21. SEM images of A/C-V_2O_{3-x}@C-HMCS after 1000 cycles.

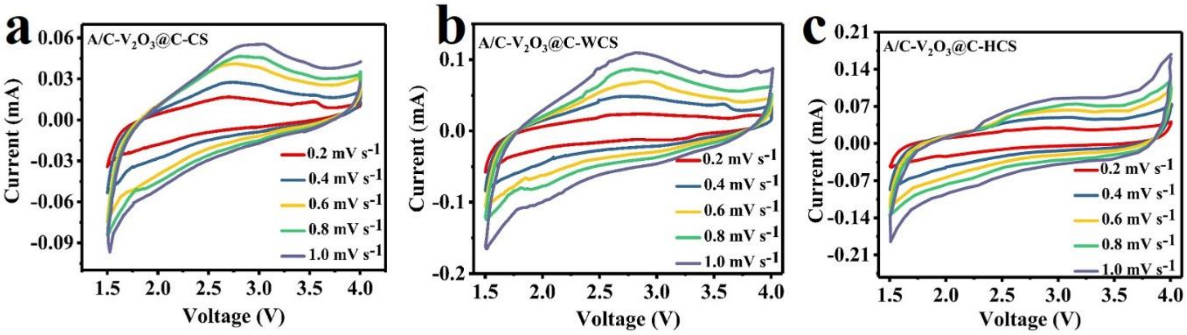

Figure S22. CV profiles at different scan rates (0.2-1.0 mV s^{-1}) between 1.5-4.0 V (versus Na^+/Na) of A/C-V_2O_3@C-CS, A/C-V_2O_3@C-WCS, and A/C-V_2O_3@C-HCS cathodes.

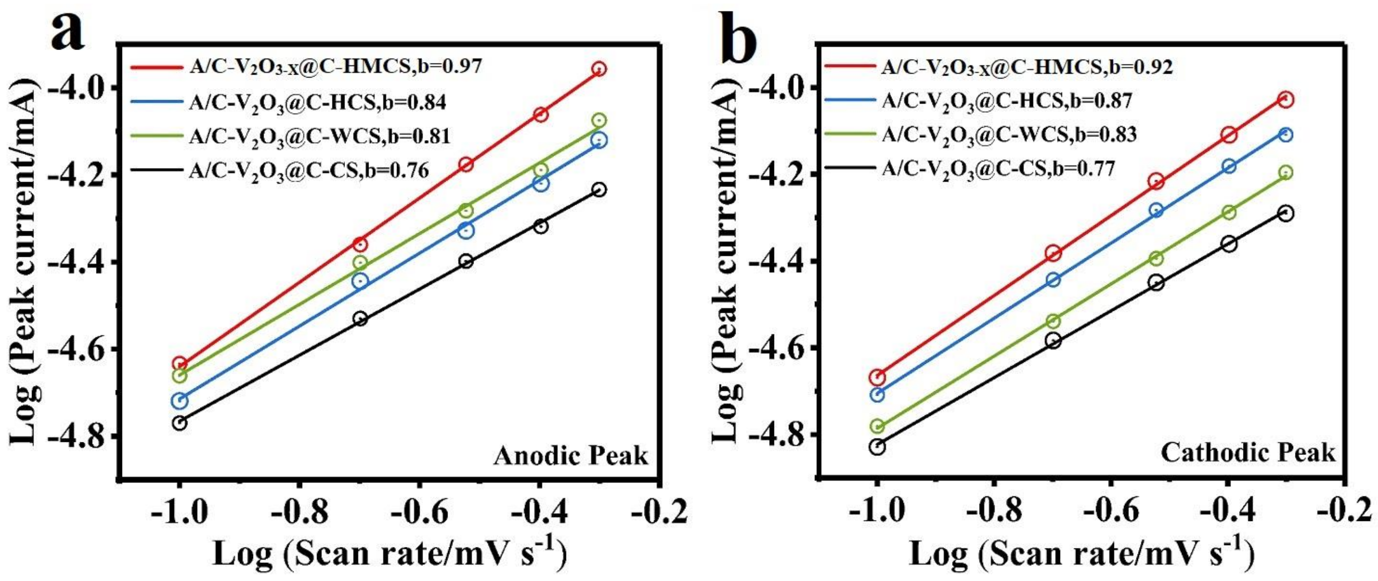

Figure S23. Calculated b-values from log (peak current) versus log (scan rate) plots for A/C-V_2O_3@C-CS, A/C-V_2O_3@C-WCS, A/C-V_2O_3@C-HCS, and A/C-V_2O_{3-x}@C-HMCS.

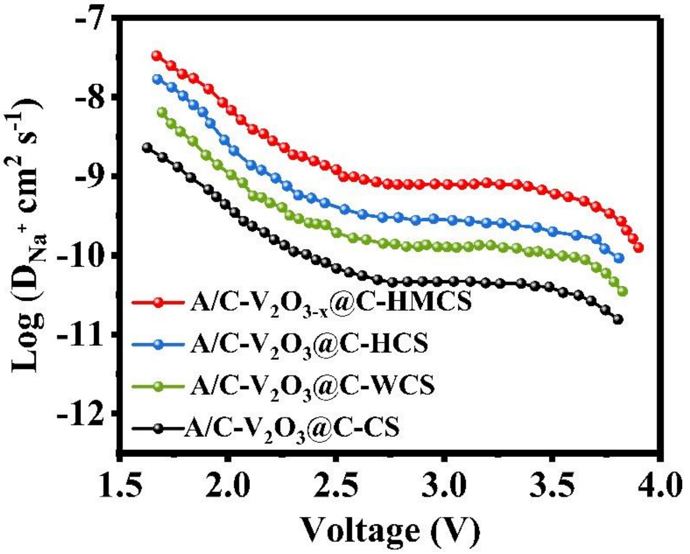

Figure S24. Diffusion coefficients calculated from the GITT during the desodiation process of A/C-V_2O_3@C-CS, A/C-V_2O_3@C-WCS, A/C-V_2O_3@C-HCS, and A/C-V_2O_{3-x}@C-HMCS.

REFERENCES*

[1] S. Osman, S. Zuo, X. Xu, J. Shen, Z. Liu, F. Li, P. Li, X. Wang, J. Liu, *ACS Appl. Mater. Interfaces* **2021**, 13, 816.

[2] S. Osman, C. Peng, J. Shen, F. Li, W. Huang, J. Liu, J. Liu, D. Xue, M. Zhu, *Nano Energy* **2022**, 107481.

[3] S. Tepavcevic, H. Xiong, V. R. Stamenkovic, X. Zuo, M. Balasubramanian, V. B. Prakapenka, C. S. Johnson, T. Rajh, *ACS Nano* **2012**, 6, 530.

[4] Y. Li, C. Liu, Z. Xie, J. Yao, G. Cao, *J. Mater. Chem. A.* **2017**, 5, 16590.

[5] H. Huang, T. Tian, L. Pan, X. Chen, E. Tervoort, C.-J. Shih, M. Niederberger, *J. Mater. Chem. A.***2019**, 7, 16109.

[6] B. Yan, X. Li, Z. Bai, L. Lin, G. Chen, X. Song, D. Xiong, D. Li, X. Sun, *J. Mater. Chem. A.* **2017**, 5, 4850.

[7] Q. Pang, Y. Zhao, Y. Yu, X. Bian, X. Wang, Y. Wei, Y. Gao, G. Chen, *ChemSusChem* **2018**, 11, 735.

[8] X. Song, J. Li, Z. Li, Q. Xiao, G. Lei, Z. Hu, Y. Ding, H. M. Kheimeh Sari, X. Li, *ACS Appl. Mater. Interfaces* **2019**, 11, 10631.

[9] Y. Fang, Q. Liu, L. Xiao, Y. Rong, Y. Liu, Z. Chen, X. Ai, Y. Cao, H. Yang, J. Xie, C. Sun, X. Zhang, B. Aoun, X. Xing, X. Xiao, Y. Ren, *Chem* **2018**, 4, 1167.

[10] Y. Cai, J. Zhou, G. Fang, G. Cai, A. Pan, S. Liang, *J. Power Sources* **2016**, 328, 241.

[11] Y. Dong, S. Li, K. Zhao, C. Han, W. Chen, B. Wang, L. Wang, B. Xu, Q. Wei, L. Zhang, X. Xu, L. Mai, *Energy Environ. Sci.* **2015**, 8, 1267.

[12] R. Gao, R. Tan, L. Han, Y. Zhao, Z. Wang, L. Yang, F. Pan, *J. Mater. Chem. A.* **2017**, 5, 5273.

[13] J.-Z. Guo, P.-F. Wang, X.-L. Wu, X.-H. Zhang, Q. Yan, H. Chen, J.-P. Zhang, Y.-G. Guo, *Adv. Mater.* **2017**, 29, 1701968.

[14] J.-Z. Guo, A.-B. Yang, Z.-Y. Gu, X.-L. Wu, W.-L. Pang, Q.-L. Ning, W.-H. Li, J.-P. Zhang, Z.-M. Su, *ACS Appl. Mater. Interfaces* **2018**, 10, 17903.

[15] Y. Cai, X. Cao, Z. Luo, G. Fang, F. Liu, J. Zhou, A. Pan, S. Liang, *Adv Sci (Weinh)* **2018**, 5, 1800680.

[16] S. Li, X. Song, X. Kuai, W. Zhu, K. Tian, X. Li, M. Chen, S. Chou, J. Zhao, L. Gao, *J. Mater. Chem. A.* **2019**, 7, 14656.
